# J. J. Gibson’s “Ground Theory of Space Perception”

**DOI:** 10.1177/20416695211021111

**Published:** 2021-06-30

**Authors:** H. A. Sedgwick

**Affiliations:** 14630 SUNY College of Optometry, New York, United States

**Keywords:** 3D perception, contours/surfaces, cue combination, depth, perception, perception/action, scene perception, texture

## Abstract

J. J. Gibson's ground theory of space perception is contrasted with Descartes’ theory, which reduces all of space perception to the perception of distance and angular direction, relative to an abstract viewpoint. Instead, Gibson posits an embodied perceiver, grounded by gravity, in a stable layout of realistically textured, extended surfaces and more delimited objects supported by these surfaces. Gibson's concept of optical contact ties together this spatial layout, locating each surface relative to the others and specifying the position of each object by its location relative to its surface of support. His concept of surface texture—augmented by perspective structures such as the horizon—specifies the scale of objects and extents within this layout. And his concept of geographical slant provides surfaces with environment-centered orientations that remain stable as the perceiver moves around. Contact-specified locations on extended environmental surfaces may be the unattended primitives of the visual world, rather than egocentric or allocentric distances. The perception of such distances may best be understood using Gibson's concept of affordances. Distances may be perceived only as needed, bound through affordances to the particular actions that require them.

## The Ground Theory of Space Perception

The theoretical and experimental study of visual space perception has, since the middle of the 20th century, been profoundly affected by the work of J. J. Gibson. The experimental paradigms and problems that he helped to establish continue to be widely used and investigated today. The fate of his theoretical work has been more mixed; some of his ideas have been widely accepted, but others have proved to be controversial—accepted or developed enthusiastically by some, while being rejected or ignored by others. What has perhaps received less attention from either Gibson’s supporters or his detractors is the degree to which Gibson’s own thinking changed over the several decades during which he developed his ideas.

In 1950, when J. J. Gibson published what he called the “ground theory of space perception,” he described it as “a hypothesis with a vast set of new implications” (Gibson, 1950a, p. 6). It is that hypothesis, and its subsequent development, that is discussed in this paper. An underlying theme of this discussion is that an idea that is radically new—as Gibson^1^ believed his to be—can take a long time, and the efforts of many people, to be fully worked out (cf. Kuhn, 1962). The “ground theory,” I will suggest, is still a work in progress and still offers a potentially rich field of investigation.

### The Cartesian Theory of Space Perception

To understand why Gibson believed his approach to be radically new, we need to look briefly at the theoretical approach to space perception that he wanted to replace. In 1950, that approach, in various forms, had been prevalent for over 300 years. A clear formulation of it was published by René Descartes in 1637, in his essay on “Optics,” so I will refer to it here as the “Cartesian theory” of space perception (Descartes, 1637/1965).

In mathematics, Descartes is remembered primarily as the inventor of analytic geometry, which applied algebra to the analysis of geometric forms, and the “Cartesian coordinate system,” which mathematicizes space by specifying the location of any point within a three-dimensional (3D) space in terms of its location relative to three orthogonal axes (Hatfield, 2018).

Descartes applied this same approach to visual space perception, although here he implicitly used a spherical coordinate system, in which the position of any point in space can be specified by its angular direction and its distance from the eye of the observer. The 3D sizes and shapes of objects were reduced to the directions and distances of the points that composed them. Descartes wrote:

As to the manner in which we see the size and shape of objects, I need not say anything in particular, inasmuch as it is all included in the manner in which we see the distance and the angular direction of their parts. (Descartes, 1637/1965, p. 107)^2^

Furthermore, anticipating by two centuries Johannes Müller's Law of Specific Nerve Energies, Descartes asserts that the angular direction of a point is determined by the particular (nerve) fiber at the back of the eye that is stimulated by the light from that point (Descartes, 1637/1965, p. 101). Thus, by a compellingly powerful and thoroughly reductive analysis, Descartes reduced all of space perception to the problem of determining the distance from the eye of a point in space.

### The Airplane Pilot’s Space

During the Second World War, Gibson led a U.S. army air force research group investigating the very practical problem of space perception in pilots flying and landing airplanes, and the associated problems of selecting and training pilots to be proficient in these critical tasks. At the end of the war, Gibson produced a book-length government report, *Motion Picture Testing and Research*: *Report No.7*, summarizing the research done by his group (Gibson, 1947); this report contains the kernel of his developing ground theory.^3^

Only about 10 pages of Gibson’s 1947 report are devoted to his new theory, and those pages are focused on the tasks at hand, but nevertheless, two basic premises of the ground theory are there explicitly, and much more is implicit. Gibson begins, reasonably enough, by saying that to effectively test prospective pilots’ space perception abilities, the researchers need to understand space perception. After arguing that the traditional theoretical emphasis on binocular stereopsis is inappropriate for space perception over the large distances of aviation, and that “monocular cues” are an undeveloped and heterogeneous list that has “not been brought together into a consistent theory explaining how they can function,” he undertakes to “define them and formulate a theory.” He then lays out his first premise, on the “continuous terrain,” which is given here in a series of quotes from his report (Gibson, 1947):

Conceiving the problem in the traditional way, distance perception in general consists of the ability to judge the distances of a number of specific objects. This, however, is not the space in which the pilot flies. What he perceives is a continuous space. It is almost never a single distance that he needs to judge, but a dimension of distance. There is invariably beneath him a continuous terrain, and what he discriminates is the location of all points on this terrain rather than the specific distances of given points … .[T]he theory behind [this] should be a theory of continuous space with an underlying terrain in which the observer is himself located and in which he can move. (pp. 184–185)

The problem of three-dimensional vision, or distance perception, is basically a problem of the perception of a *continuous surface* which is seen to extend away from the observer … .[A]n array of objects by themselves does not make up visual space; it is constituted instead by the ground or surface against which these shapes and figures appear. The visual world consists of object-surfaces on a background of an extended ground surface. (pp. 185–186)

We need to explain not the “cues” or “indicators” to the distances of specific objects but instead the dimension or sensory continuum of distance, *as such*, which, once visible, determines how distant all the objects within it are … .If this view is correct, it is necessary to see a continuous surface in order to have an accurate sense of continuous distance. (p. 186)

**Figure 1.**
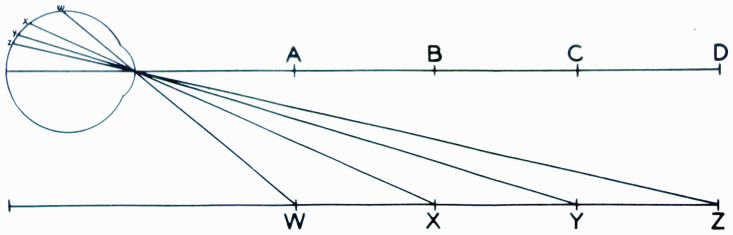
“Two formulations of the problem of distance perception.” (Gibson's caption. From Gibson, 1947.)

Gibson sharply contrasts his new theory with what I am calling the Cartesian theory, but which he simply calls the “traditional way” or the “classical formulation.” He includes a diagram (Figure 1) that succinctly captures his argument:

**Figure 2.**
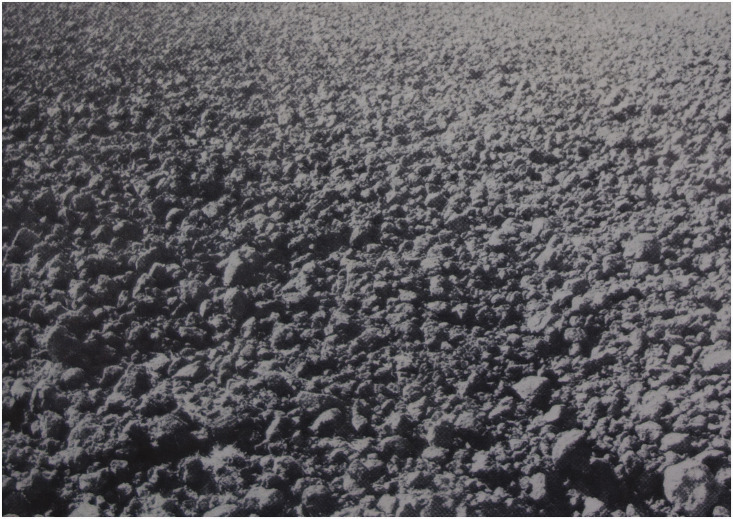
“Distance as produced by a natural gradient of texture.” (Gibson's caption. From Gibson, 1947.)

**Figure 3.**
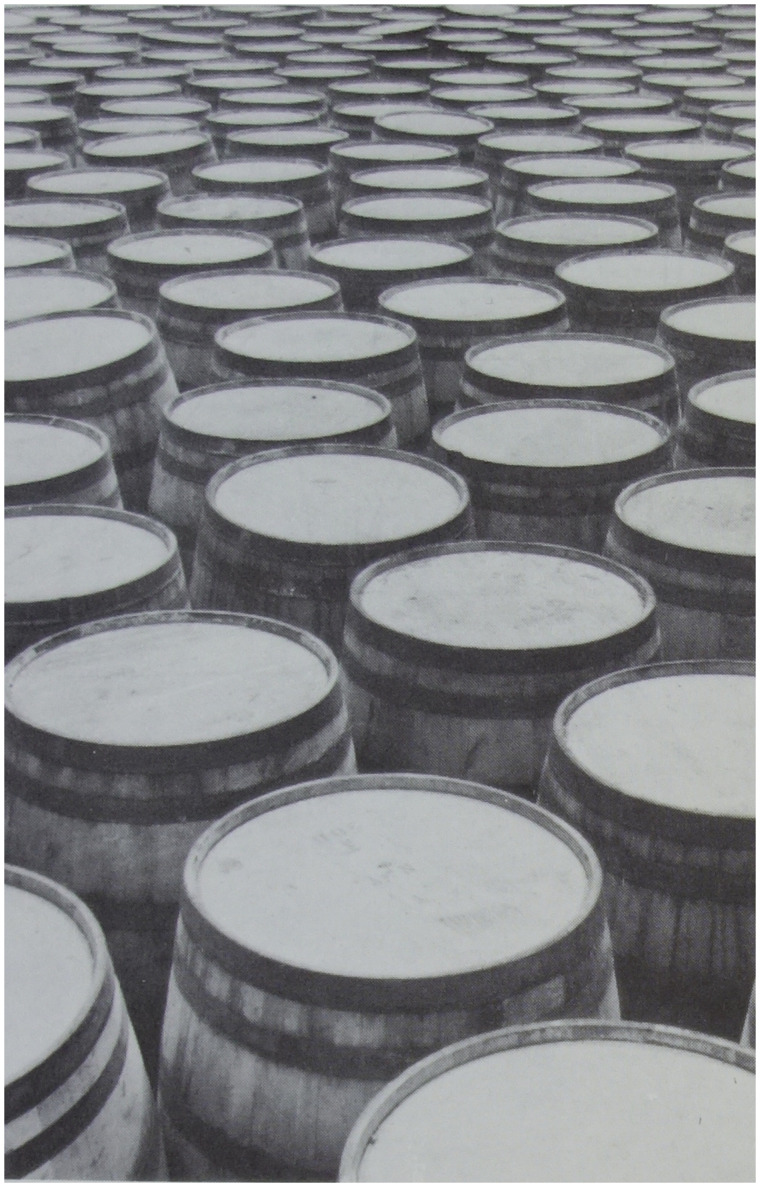
“The perspective of objects is the same in principle as the perspective of texture.” (Gibson's caption. Adapted from Gibson, 1950a.)

This view of the problem is in contrast to the classical formulation which asks how the retina of the eye can see a third dimension in the sense of a theoretical line extending outward from the eye. Points on this line at different distances must all be identical so far as the retina is concerned. Nevertheless, we do see depth. How can this be? The solution to this dilemma is to recognize that visible distance does not consist of a line extending outward from the eye. The question to ask is not how do we see such a line but how do we see the substratum—the surface which extends away from us in the third dimension? The image of this surface is obviously *spread out* across the retina

Figure 1 illustrates the two formulations of the problem. The points A, B, C, and D cannot be discriminated by the retina. Distance along this line is a fact of geometry but not one of optics or of visual perception. But the points W, X, Y and Z at corresponding distances can be discriminated by the retina. They represent the retinal image which corresponds to an extended substratum. (Gibson, 1947, p. 186)

One way that the Cartesian problem of depth perception is often expressed is to say something like the following:

Space is a three-dimensional volume but the retina is only a two-dimensional surface, so in the process of projection that forms an image on the retina, one dimension is lost; that lost dimension is depth. That loss is mathematically demonstrable and is, in the deepest sense, irreparable; fundamentally, the problem of visual depth perception is insoluble. The cues to depth, such as elevation in the visual field, are only workarounds; they can help us guess what that lost depth might have been, but they are fallible, and vision in fact often fails. In the most basic case—a single eye looking at a point of light in empty space—the perception of the spot’s distance bears almost no relation to its true distance

The first and most fundamental step of Gibson’s new theory, is to resolve this dilemma of the lost depth dimension by pointing out that people do not live in an abstract empty space; they live on (or above, in the case of airplane pilots) the surface of the earth. Images of extended surfaces, such as the terrain of the earth, are spread out on the retina; different distances along those two-dimensional surfaces are imaged in a one-to-one mapping onto the two-dimensional retina and so are available to visual perception. A two-dimensional environmental surface has been mapped onto the two-dimensional retinal surface. *Nothing has been lost*. Thus, the whole apparatus of “cues” for recovering the lost depth is unnecessary. The mystery of how depth could be added to an image point on the retina has disappeared.

### Ecological Constraints

In 1950, describing his wartime research Gibson wrote: “Experiments needed to be performed *outdoors*. The stimuli to be judged ought to be those of a *natural environment*” (Gibson, 1950a, p. 6).

Although Gibson did not suggest the terminology of “ecological optics” until 1957, his 1947 concept of the continuous terrain is already ecological.^4^ The observer is not just an abstract eye in empty space but is embodied and is situated in a natural environment. The most basic situation for Gibson is a person standing on a ground terrain—a continuous textured surface—that extends to the horizon.

The terms “ecological” or “natural” imply constraints. The ground is more or less horizontal, and it supports the observer, and other objects, against the pull of gravity. This scene is lit by sunlight coming from above. These are not the characteristics of any conceivable space, but instead are the characteristics of the natural environment that we, as people, inhabit and to which we, and our perception, are adapted.

These constraints transform the “problem” of space perception. For Cartesian theory, if the retina is stimulated by light coming from a particular direction, then the corresponding point in empty space could be at any distance along that particular direction. But what is infinitely ambiguous for an observer in empty space becomes unambiguous for an observer in their natural environment. Gibson presented and discussed Figure 1 again in 1950, calling the Cartesian problem of distance perception a false problem (Gibson, 1950a, p. 9).

### Gradients of Stimulation

The second theoretical premise introduced in Gibson’s 1947 *Research Report*^5^ concerns the stimulation that produces the perception of a continuous surface. When the image of an extended environmental surface is projected onto the retina, *instead of loss*, *there is transformation*; the spatial characteristics of two-dimensional surfaces in the environment are optically transformed into the complex characteristics of two-dimensional retinal images—characteristics such as gradients of texture, motion perspective, and binocular disparity that are often in one-to-one correspondence with the characteristics of the environmental surfaces. According to Gibson’s *Research Report*, it is these characteristics of the retinal image that are then the stimuli for perception.

Again anticipating ecological optics, Gibson presents the characteristics of the retinal image as a necessity arising from the characteristics of physical surfaces and from optics: “The retinal image of the surface must differ significantly at different points corresponding to those which are farther or nearer. There must, in other words, be retinal *gradients* of stimulation” (p.186, my underlining). This premise is then the basis for a reformulation of the “cues” for distance in terms of gradients. Gibson briefly describes five types of retinal gradients:

1. The retinal gradient of texture (Figure 2). Gibson references Metzger and Koffka as showing that “the difference between the perception of a surface, such as a wall, and the perception of an area without surface, such as the sky” is that the perception of “surface corresponds to a retinal image having minute irregularities … ,” which they refer to as microstructure. Speaking of gradients, Gibson says that “the retinal image may vary between extremely coarse and extremely fine differentiation. In order to include the extremes of this stimulus variable, it will here be called not microstructure but ‘texture’” (pp.188–189). Referring to any textured surface, such as the ground, Gibson writes:
  If it extends away from the observer, the retinal texture becomes finer as the distance of the corresponding points of the surface becomes greater … There will exist a continuous gradient of texture from coarse to fine with increasing distance of the surface … It may be noted that the stimulus-correlate of distance … is not the gross retinal size of the texture-elements but their relative size within the gradient. (p. 189)
  Gibson distinguishes his illustrations of texture gradients from linear perspective because they do not contain straight lines converging to the horizon. But he goes on to say
  The texture gradient is, however, a kind of perspective in the broad sense of that term and it is related to linear perspective inasmuch as in the case of both variables retinal size decreases with distance and vanishes at the horizon. All the retinal gradients to be described as stimulus variables for distance perception are analogous to perspective … The variable just described, therefore, might well be given the name of texture perspective. (p. 191)
2. The retinal gradient of size-of-similar-objects, by which Gibson means classes of similarly sized objects, such as houses, or telephone poles, or people. For each such class of objects, if there are enough of them located at various distances in the environment, they can form a gradient of retinal sizes that decrease with distance (Figure 3).
3. The retinal gradient of velocity during movement of the observer, which is a “continuous gradient of motion of the ground” that Gibson calls “motion perspective” (p. 192).
4. The retinal gradients arising from atmospheric transmission of light, which is the monocular cue of aerial perspective applied to a natural terrain.
5. The retinal gradient of binocular disparity between the retinal images of the two eyes. This gradient of binocular disparity along the ground is described as being greatest at the foot of the observer and decreasing to zero at the horizon.

With these two premises—replacing depth perception with surface perception and replacing cues to depth with gradients of retinal stimulation—Gibson laid the groundwork for a new theory of visual perception.^6^

### The Visual World and the Visual Field

Following the end of the war, Gibson returned to his pre-war position at Smith College and immediately began writing a book on visual perception (Gibson, 1967). In 1949, he moved to Cornell University where, during his first year there, *The Perception of the Visual World* was published (Gibson, 1950a). In this book, Gibson presented an exposition of his novel theoretical concepts, and he explored the application of these new concepts to a wide range of topics in visual perception. What originated in the imperatives of a wartime research project on flying airplanes is enlarged in the *Visual World* to encompass all of visual perception.

Although the phrase *visual world* occurs in his 1947 *Research Report*, Gibson now develops it as a theoretical concept, contrasting it with his concept of the *visual field.*

A substitute for the distinction between sensation and perception will be offered … , a substitute intended to retain what is verifiable in the classical distinction and eliminate what has been theoretically misleading. We can attend either to color-impressions or to object-impressions, generally speaking. Introspection of the first sort yields an experience of the visual *field*. Introspection of the second sort, called “phenomenological,” yields an experience of the visual *world*. Both these kinds of experience must be accounted for if we are to understand vision, but the latter is the subject of this book. (Gibson, 1950a, p.11)

Gibson introduces this pair of concepts both to replace the traditional concepts of “sensation” and “perception” and to reverse their order of epistemological priority. Thus, in the traditional view, visual stimuli can be described as a pointillistic field of spots of light, of various intensities and spectral distributions, spread out across the retina; these stimuli produce sensations of a flat field of meaningless points of variously colored light, and these sensations are all that is immediately available to vision; perceptions of meaningful objects, places, and events are built up from these sensations by complex internal processes of calculation, or by association with past experiences, or in the case of the newer Gestalt psychology, by internal processes of organization.

The psychophysics of his time, as Gibson saw it, was a collection of experimental methodologies used to investigate quantitatively the connections between stimulus energies in the light reaching the retina and the elementary sensations that these stimuli produced. It was of course presumed that complex neural processes underlay the connections between stimuli and sensations, but psychophysics provided scientifically sound methodologies for uncovering the lawful relations in these connections even though the underlying neural processes were only very partially understood.

In Gibson’s view, the fundamental error of the existing psychophysics was a failure to recognize that the light reaching the eye has already been organized into complex structures by its interaction with the environment. He proposed four related ideas: (a) there are such structures in the light falling on the retina; (b) these optical structures are often in a 1:1 correspondence with the environmental structures that produced them; (c) in response to such stimuli, the visual system produces percepts of those environmental structures; and (d) the correspondences between these complex stimuli and the resulting percepts can be studied using the methodologies of psychophysics. In the *Visual World*, Gibson encompassed all of these ideas with his hypothesis of *psychophysical correspondence*, which he saw as extending psychophysics from the study of sensation to the study of perception. Doing so provided him with a rigorous methodology to investigate his ground theory.

**Figure 4.**
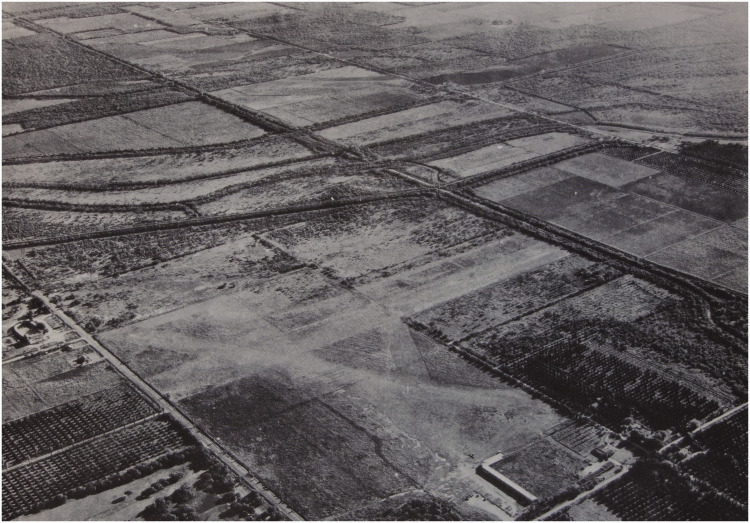
“The look of the world from the air.” (Gibson's caption. Adapted from Gibson, 1950a).

In the *Visual World*, Gibson greatly developed and expanded on the premises in his 1947 *Research Report.* It was here that he named his ‘ground theory’ and connected it with his concept of the visual world (Figure 4).

He proposed:

A hypothesis …  *that there is literally no such thing as a perception of space without the perception of a continuous background surface*. This hypothesis might be called a “ground theory” … The basic idea is that visual space should be conceived not as an object or an array of objects in air but as a continuous surface or an array of adjoining surfaces. The spatial character of the visual world is given not by the objects in it but by the background of the objects … .It is exemplified by the fact that the airplane pilot’s space, paradoxical as it may seem, is determined by the ground and the horizon, not by the air through which he flies. (Gibson, 1950a, p. 6)

It is important to note here that although Gibson’s ground theory is named for, and exemplified by, the importance of the ground in visual space perception, the theory is not exclusively concerned with the surface of the ground. In the *Visual World*, he applies it more generally to all the background surfaces that, according to Gibson, provide the “spatial character of the visual world.” These continuous background surfaces may have any spatial orientation. In a complex visual world, such background surfaces can form “an array of adjoining surfaces,” as with the floor, walls, and ceiling of a room.^7^

In *Visual World*, gradients remain central to Gibson’s description of the environmentally produced retinal stimuli that shape the perception of the visual world. As in his *Research Report*, he relates all of these gradients to perspective:

The sensory impressions which go with the perceptions of distance or depth over a continuous surface might all be called varieties of perspective …  [They] correspond with gradients of adjacent stimulation on the retina …  The varieties of perspective can be listed somewhat as follows: 1. Texture-perspective …  2. Size-Perspective … 3. Linear Perspective …  4. Binocular perspective …  5. Motion-perspective …  6. Aerial perspective …  7. The perspective of blur …  8. Relative upward location in the visual field … . (Gibson, 1950a, pp. 138–141)

In his discussion of this list, Gibson gives the status of “stimulus” only to the first five; the remaining three are downgraded in various ways. For example, of aerial perspective he says that it is likely to be only “an indicator” of distance. Also, the first three on the list can be grouped under one heading, in which size perspective and linear perspective are subsumed under texture perspective. Size perspective is described as a distribution of similarly sized objects that are treated visually as a texture; an example that Gibson gives later (p. 85) is the trees in a forest, which at one distance are seen as individual objects, but at a greater distance appear as the texture of the forest. Linear perspective is described as a special case of texture gradients in which the texture elements are regularly arranged, rather than irregularly. Throughout the *Visual World*, Gibson’s emphasis is thus on the three gradients of texture perspective, motion perspective, and binocular perspective, which are sometimes referred to as gradients of density, deformation, and skew, respectively. But gradients become only an example of the broader concept of “higher-order variables” and the hypothesis of psychophysical correspondence: “There is always some variable in stimulation (however difficult it may be to discover and isolate) which corresponds to a property of the spatial world” (p. 8).

Gibson sees his hypothesis of psychophysical correspondence as complementary to his ground theory. The ground theory hypothesizes that the structure of the retinal image is in one-to-one correspondence with the structure of the visible surfaces of the environment; psychophysical correspondence is the hypothesis that surface perception is then in one-to-one correspondence with the retinal stimulation that produces it.

Inherent in Gibson's application of psychophysical correspondence to the perception of the environmental layout of surfaces is his idea that any surface is perceived from the first as having a 3D orientation. This perception is an unmediated response to the gradients in the retinal stimulus. If a surface is receding in depth, then its projected texture has a perspectivally produced gradient of density on the retina that produces the perceptual response of a receding surface. If a surface is in the frontal plane, then its projective gradient of texture density is zero and produces the perceptual response of a frontal surface. There is nothing special about the perception of a frontal surface. In both cases—frontal or receding—retinal stimulation produces a perceptual response. Specifically, in the process leading to a perception of a receding surface, there is never a perceptual stage before depth has been added to it; that is, there is never a stage in which the surface is registered as a frontal form, lacking in depth. This is the sense in which Gibson later said that the gradient of texture density leads *directly* to the perception of a slanted surface.

As a corollary to this theoretical structure, percepts no longer needed to be built up from sensations by processes of internal organization; that organization was already present in the stimuli. Sensations now became secondary; they were rarely noticed in everyday life, but they could be experienced to some limited extent by careful introspection, by paying attention to visual angles and small patches of color as a painter might do. It was these experiences that Gibson described as the *visual field*.

### From Retinal Stimulation to the Optic Array

In Gibson’s preface to *The Senses Considered as Perceptual Systems* (Gibson, 1966), he looks back at the period from the late 1950s to the early 1960s:

This book has had to be written twice—once in 1958–59 and again in 1963–64. The second draft is more explicit and coherent than the first, for a good deal of experimenting, teaching, arguing, reading, and reformulating came in between … It was difficult to shake off the traditional explanations of the facts while keeping hold of the facts themselves. (Gibson, 1966, p. vii)

Because Gibson’s ground theory was the focus of much of his early work, before the reformulations that he refers to here, it is helpful to look at these conceptual developments before looking in more detail at the development of the ground theory.

In his 1947 *Research Report*, Gibson describes gradients as types of retinal stimulation necessary to produce the perception of a continuous surface. There are complications in describing these gradients in terms of retinal stimulation, however, because, as Gibson knew, the eyes move several times a second. With the optical projection of a surface possibly extending over a substantial portion of the visual field, the temporal sequence of retinal stimulations arising from eye movements could be complex.

For example, a target's binocular retinal disparity is zero when the target is at the fixation point to which the two eyes are converged. This binocular retinal disparity increases as the target is moved either nearer or farther from that fixation point. In Gibson’s perspective-based analysis of gradients, however, binocular disparity is greatest when the target is closest to the observer and decreases to zero when the target is at the horizon. To resolve this discrepancy, Gibson defined the binocular disparity gradient in terms of relative disparity rather than retinal disparity: “The stimulus which is concomitant with distance, therefore, is not simply disparity as such but disparity relative to a gradient … ” (Gibson, 1947, p. 194). This relative gradient of binocular disparity is independent of the amount of convergence of the observer’s eyes, and so is awkward to describe in terms of retinal stimulation.

An analogous complication arises with motion perspective. Gibson describes the gradient of motion perspective as being maximal directly beneath the observer and decreasing to zero at the horizon. This would only be true of retinal stimulation, however, if the eye were fixating a location on the horizon. If the eye tracks a ground location at some intermediate distance, then the angular vector of eye rotation is subtracted from all retinal motions, bringing the retinal motion of the tracked location down to near zero, decreasing the retinal motions of ground locations nearer than the tracked location and producing retinal motion in the opposite direction for farther ground locations. Gibson’s solution to this complexity is again to define the motion perspective gradient as being based on relative motion rather than absolute retinal motion. He writes that

[t]he gradient of retinal velocities with respect to their direction is therefore unaffected by pursuit movement of the eyes … We must suppose that the effective stimulus for such perception is the gradient of velocities in the retinal field—the direction and rate of change along a retinal axis—rather than the velocities themselves. (Gibson, 1947, p. 225)

In the *Visual World*, Gibson explicitly loosens the tight connection between retinal activity and the perception it is hypothesized to produce. In a section titled “The Retinal Image and the Excitation of the Retinal Mosaic,” he writes,

It is easy to assume that the retinal image and the retinal excitation are the same thing. But the former, clearly, is a matter of physics while the latter is a matter of physiology. The image is an arrangement of light-points while the excitation is an arrangement of discharging nervous elements. (Gibson, 1950a, p. 55)

Gibson is separating the image optically projected onto the retina, as if onto a screen, from the excitation that this image produces. A striking effect of this separation is that the retinal image is now tied to the world instead of to the physical retina. “Above all,” Gibson writes,

since the image is an event in the light-flux of the physical world, it has reference to the world and is fixed in relation to it. It keeps a constant alignment with gravity, for instance, when the head is tilted and the retina rotated. (Gibson, 1950a, p. 55)

This is an important anticipation of Gibson's concept of the optic array.

What Gibson initially called the “optical array” is clearly described in a 1959 chapter that systematically presents his theory.^8^ He writes that “[t]he optical stimulus can be analyzed at any of several stages or levels of abstraction before the excitation of receptors.” Immediately prior to retinal excitation is the retinal image, and at a level prior to that “there is the whole array of focusable light converging to any given location in the open air. This may be termed the optical array, one sector of which is picked up by an eye.” A little farther on, he writes, “The various aspects of pattern can be specified on the retina with some difficulty, on a hypothetical ‘picture plane’ with less difficulty, and in the optical array very simply, since angular coordinates can be employed” (Gibson, 1959, pp.472–474).

The concept of the optic (or optical) array gave Gibson a framework within which one could investigate relations between perception and the structure of light reaching the eye without having to include the study of eye movements in that investigation.

### From Psychophysical Correspondence to Potential Stimulus Information

Although Gibson’s concept of the optic array freed his theory from the need to shoehorn his higher-order variables into the retinal image, his 1959 chapter held firmly to his idea of psychophysical correspondence.^9^ Under the heading “The generalized hypothesis of psychophysical correspondence,” he writes: “Perception is said to be a function of stimulation. This means that it is exclusively a function of stimulation whenever the conditions of stimulation permit” (Gibson, 1959, p. 465, my underlining).

In the same 1959 paper, Gibson outlines two stages in what he calls “[t]he chain of causation leading to perception”:

The chain of causation can be considered in two parts—that outside the organism and that inside. A complete theory of perception must deal with both, but each should be made separately explicit. The first part is concerned with the biophysics of stimulation, that is, the nature of the environment and the relation between object and stimulus. The second part is concerned with the variables and properties of stimulation and the relation between these and perception or what can be called the psychophysics of perception. (p. 464)

He goes on to link these two stages together: “[t]he outline has been presented of what might be called a psychophysical theory of perception joined to a biophysical theory of stimulation” (p.473).

In 1960, Gibson was president of the Eastern Psychological Association. His presidential address was titled “The Concept of the Stimulus in Psychology” (Gibson, 1960). In this major paper, he surveys and analyzes multiple uses of the term “stimulus” in current psychological theorizing. It seems clear that he is using the occasion of this paper to carefully reexamine and develop his own ideas about what it means to be a stimulus. One development here that is of central importance to his concept of the optic array is a distinction between *potential stimuli* and *effective stimuli.* Gibson writes:

[T]he hypothesis of potential stimulation … has quite radical but unrecognized implications. We have long acknowledged the almost unlimited possibilities for new responses in learning theory; why not equally vast possibilities of new stimuli? The environment, so considered, would consist of a sort of reservoir of possible stimuli for both perception and action … The variables and covariables and invariables of this stimulus environment are inexhaustible

Surprisingly little has been written about potential stimuli … I think that we will have to develop the needed discipline on a do-it-yourself principle. It might be called ecological physics, with branches in optics, acoustics, dynamics, and biochemistry. (Gibson, 1960, pp. 700–701)

Here Gibson is introducing the concept of “ecological optics,” which is the study of the relation between the structure of the environment and the structure of the light reaching the eye. Gibson goes on:

An effective stimulus can now be defined. It is one which arouses receptor activity, or recorded neural impulses, or sense organ adjustments, or overt responses, or verbal judgments—whichever criterion one chooses

[W]hether or not a potential stimulus becomes effective depends on the individual. It depends on the species to which he belongs, on the anatomy of the sense organs, the stage of maturation, the capacities for sense organ adjustment, the habits of attention, the activity in progress, and the possibilities of educating the attention of the individual. (Gibson, 1960, p. 701)

In his 1959 article, quoted earlier, Gibson posited a two-part unified theory: “a psychophysical theory of perception joined to a biophysical theory of stimulation.” The 1960 concept of the potential stimulus marks a clear separation between these two theories, allowing these two domains to be investigated independently of each other. *Ecological optics* is a biophysical theory of *potential* stimulation. It is a field of study in its own right, separate and distinct from the psychophysical investigation of the conditions, if any, under which these *potential* stimuli become *effective*.

This distinction between a potential stimulus and an effective stimulus creates a more flexible linkage between the optic array and perception. The potential stimuli in the optic array far exceed the ability of any particular organism to respond to those stimuli. Whether a potential stimulus becomes effective perceptually depends upon the organism; it depends not only on its fixed characteristics such as its species and the anatomy of its sense organs, and not only on its long-term characteristics such as its maturation and its prior experience, but also on its moment-to-moment engagement with the world: fluctuations in its attention, it current activity, and so on. This is a significant modification of Gibson’s hypothesis of psychophysical correspondence; perception is no longer *exclusively* a function of stimulation.

The final stage of development to be considered here in the conceptual framework underlying Gibson’s ground theory is his introduction of the concept of *stimulus information*. In his article “Ecological Optics” (Gibson, 1961), he writes:

[T]he variables of an optic array may *carry information* about the environment from which the light comes. This is a central hypothesis for ecological optics. By “carry information,” I mean only that certain variables in an array, especially a moving array, will correspond to certain properties of edges, surfaces, things, places, events, animals, and the like—in short to environmental facts. They will not, of course, replicate but only specify such facts. (Gibson, 1961, reproduced on pp. 68–69 in Reed & Jones, 1982)

One purpose of this new terminology is to further distance his theory from the idea, implied by the terminology of psychophysics, that perception is a passive response imposed by environmental stimuli.

In *The Ecological Approach to Visual Perception* (Gibson, 1979), Gibson looks back at his original theory of psychophysical correspondence. What he has to say is quoted at length here because it is his own view of the conceptual developments that I have been describing:

There was to be a new psychophysics of perception as well as the old psychophysics of sensation. For I thought I had discovered that there were stimuli for perceptions in much the same way that there were known to be stimuli for sensations. This now seems to me a mistake. I failed to distinguish between stimulation proper and stimulus information, between what happens at passive receptors and what is available to active perceptual systems … .

What I had in mind by a psychophysics of perception was simply the emphasis on perception as direct instead of indirect. I wanted to exclude an extra process of inference or construction. I meant (or should have meant) that animals and people *sense* the environment, not in the meaning of having sensations but in the meaning of *detecting*. When I asserted that a gradient in the retinal image was a *stimulus* for perception, I meant only that it was sensed as a unit; it was not a collection of points whose separate sensations had to be put together in the brain. But the concept of the stimulus was not clear to me. I should have asserted that a gradient is stimulus *information*. For it is first of all an invariant property of an optic array. I should not have implied that a percept was an automatic response to a stimulus, as a sense impression is supposed to be. For even then I realized that perceiving is an act, not a response, an act of attention, not a triggered impression, an achievement, not a reflex

So what I should have meant by a “psychophysical” theory of perception in 1950 and by perception as a “function of stimulation” in the essay I wrote in 1959 (Gibson, 1959) was the hypothesis of a one-stage process of the perception of surface layout instead of a two-stage process of first perceiving flat forms and then interpreting the cues for depth (Gibson, 1979, pp. 149–150)

With this introduction to Gibson’s evolving conceptual framework as background, we will now consider the development of four specific concepts that were contained within or grew out of his ground theory. These are *scale*, *orientation*, *contact*, and *location*.^10^

## Scale

### The Traditional View: Perceived Size Depends on Perceived Distance

A striking example of Gibson’s struggle to free his new ideas from the encumbrances of the Cartesian theory can be found in the development of his treatment of the relationship between perceived size and perceived distance. In Cartesian theory, the physical size of an unfamiliar object can only be determined by combining its angular size with its distance. Although Gibson believed that the ground theory dissolved the Cartesian problem of *how* distance is perceived, in his 1947 *Research Report*, he still held firmly to the belief that space perception *depends* on distance perception, as exemplified by the belief that the accurate perception of physical size depends on the accurate perception of distance. Gibson was so secure in this belief that he carried out experiments and devised tests for would-be pilots that “measured” distance perception by actually measuring size perception and then geometrically calculating what the perceived distance “must” have been. He wrote:

If a subject is able to judge the true size of a distant unfamiliar object, he does so only because he sees the true distance of the object … .The ability to estimate the sizes of distant objects or, specifically, to match them accurately with the corresponding sizes of near-by objects is therefore indicative of the ability to estimate their distance. (Gibson, 1947, p. 197)

### Scale and Distance

The kernel of the ground theory is clearly presented in Gibson’s 1947 *Research Report*, but there is a much fuller development of that theory in his 1950 *Visual World*. There, although continuous background surfaces still mediate the perception of size, Gibson introduced the concept of *scale* as a property of such surfaces. He wrote:

[S]ize-constancy experiments … imply that the dimensions of things, large or small, are comparable at different distances … .These facts suggest that we perceive a quality in the visual world which might be called *scale.*

[T]he implicit scale of visible size is a primitive feature of perception … The size constancy of objects, in the light of this conception, is a by-product of the constant scale of the visual world at different distances. Scale, not size, is actually what remains constant in perception

The size of any particular object is given by the scale of the background at the point to which it is attached (Gibson, 1950a, pp. 180–181).

With Gibson’s concept of scale, perceived size is no longer primarily a property of individual objects; instead, perceived size is secondary to, or derived from, the underlying scale of the environment—the layout of surfaces—within which individual objects are located. Initially, this “primitive feature” of scale seems clearly to have been underlain by Gibson’s concept of “continuous distance.” But continuous distance over a continuous terrain includes distances between locations on the terrain as well as distances between locations and the observer. With the addition of the concept of “scale,” the distinction between the processes of size and distance perception begins to fade. Describing the tendency of the visual world to be approximately rigid, Gibson writes:

This constancy of size and shape also appears to hold true for the ground or the floor, and for any segment or part of the background. We shall find some evidence that it also holds for the distances between objects—the shapes of the intervening spaces … (Gibson, 1950a, p. 165)

Gibson describes an exploratory experiment that he and R. H. Henneman conducted during their wartime research:

The observer was seated at the end of a thoroughly cluttered room containing tables, cabinets, boxes, shelves, and furniture. Among these objects he had to estimate 20 specified dimensions, some being the dimensions of solid things and some being dimensions in the open air between them

He goes on to say “The distinction was not as clear as it sounds, for there was always a background surface behind any dimension.” The result was “an approximate constancy of size” (p. 183) for the distances between things as well as for the things themselves.

In *Visual World*, the perception of scale and of distance are conceptually bound up with each other. Gibson writes: “The impressions of scale and of distance are so related to one another that with increasing distance there goes an unvarying scale” (p. 181). As we shall see, Gibson later concluded that a gradient of texture independently determines the perception of distance and of size, but in 1950 this was not yet clear to him (also see Figure 14). Nevertheless, his position on the dependence of size perception on distance perception seems, at least to this reader, to have been unsettled by his introduction of the concept of scale and to be softer than the statement of an absolute dependence quoted above from the 1947 *Research Report*. There is a striking change in the way that the same key experiment is described in 1947 and then in 1950. In *Visual World*, Gibson again describes his wartime experiment on matching the sizes of objects at different distances over an extended ground terrain. In 1947, because of his assumption that physical size could only be perceived based on perceived distance, he used this size-matching experiment as an indirect measure of perceived distance. In 1950, his interpretation changes and the same experiment is now described as an experiment on size constancy; no dependence on perceived distance is mentioned (pp. 183–186).

**Figure 5.**
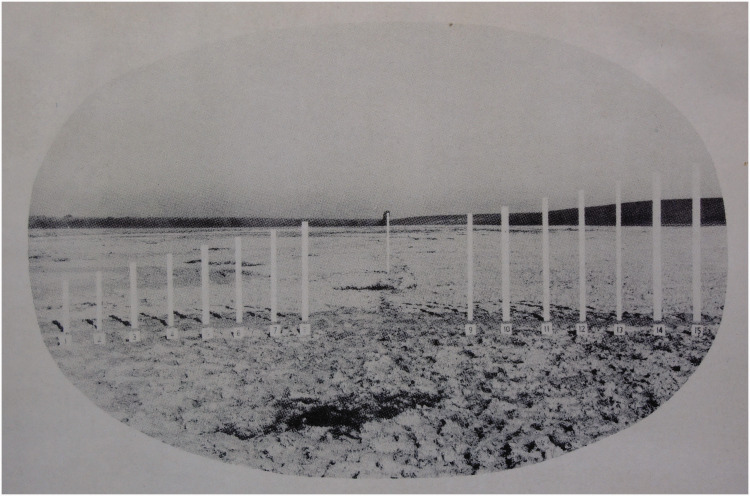
“Sample item of the distance estimation test.” In Gibson (1950a), a similar item from the same test is captioned “Judgment of size-at-a-distance.” (Phrases in quotes are Gibson's captions from 1947 and 1950a; from Gibson, 1947.)

That experiment was conducted outdoors on an extended ground terrain. Observers chose which of a series of nearby vertical poles was most similar in height to a single pole that was farther away (Figure 5). As the single pole was presented increasingly far away, the perceptual matches became more variable, but even when the single pole was so far away that it could barely be seen, there was no tendency to underestimate its physical size. Gibson concludes that size constancy applies over the whole extent of the ground terrain. Objects get harder to see as they get farther away, but they do not look physically smaller.

### Texture Scale

In the formulation that Gibson offers in 1959, which is based on what he calls textural “numerosity,” there is no difference between how the sizes of objects and the distances between them are perceived.

The perceived length of a *stretch* of distance along the ground from “there to there,” at whatever distance the stretch may lie, probably depends on the number of transitions or texture elements in the stretch relative to the number in the total range of visible distance. Note that a ratio of this sort is invariant for different textures of the ground (grass, pavement, bushes) (Gibson, 1959, p. 475).

For empirical support, Gibson refers to an experiment (Purdy & Gibson, 1955) on fractionation of perceived distance over the ground. He writes:

The same reasoning should apply to stretches of “width” along the ground at different distances as to stretches of “depth,” that is, to the frontal as well as the longitudinal dimension of a receding surface. (p. 475)

… *the size of an object is given by the size of its projection relative to the size of the elements of texture or structure in the adjacent optical array*. The stimulus for perceived size is a ratio rather than a simple magnitude … Size is perceived relative to the size scale of the place where the object is seen. (p. 479)

**Figure 6.**
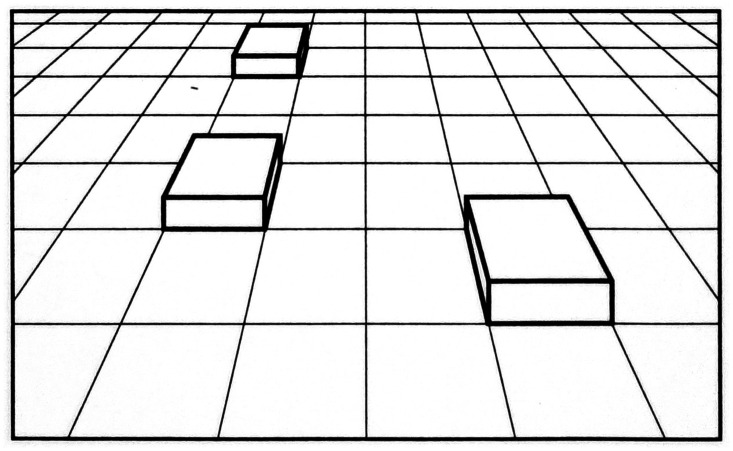
Size scale provided by ground texture. (Adapted from Sedgwick, 2001b.)

This formulation is illustrated in Figure 6, which is a schematic drawing of objects resting on a textured ground. The size of each object, relative to the other objects, can readily be perceived from the amount of texture that each object covers at its base.

In this formulation, perceived size does not depend on perceived distance, but they still go together because both are subsumed under the concept of scale. If there is a failure of the scale constancy of a particular space, there will be a failure of constancy of all the dimensions of that space (p. 479).

Gibson’s development of the concept of texture scale thus extends his rejection of the Cartesian theory; the perception of a spatial layout of surfaces no longer depends upon the perception of distance from the observer.

### Horizon-Ratio Scale

There is some question, looking at Figure 5, whether the texture elements were sufficiently visible to be useful to observers, especially when the pole’s distance was large. This led me, when I was studying with Gibson, to wonder whether there might be additional information, related to the ground plane, that could specify the height of the poles. And indeed there is. It comes from the horizon (Sedgwick, 1973).

**Figure 7.**
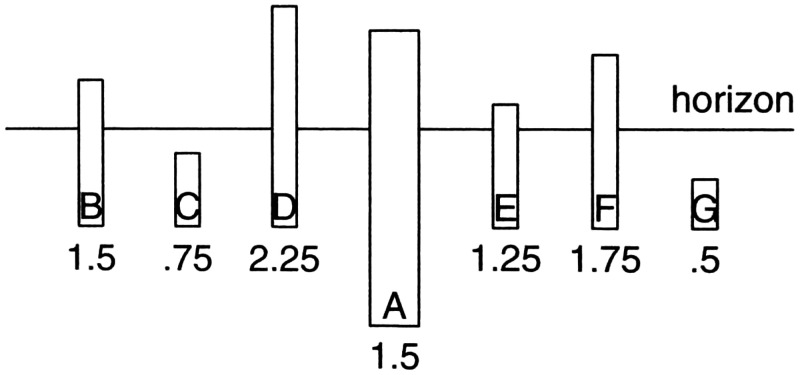
Which stick is the same height as A? The answer is B, which has the same horizon-ratio as A. (Adapted from Sedgwick, 2010.)

**Figure 8.**
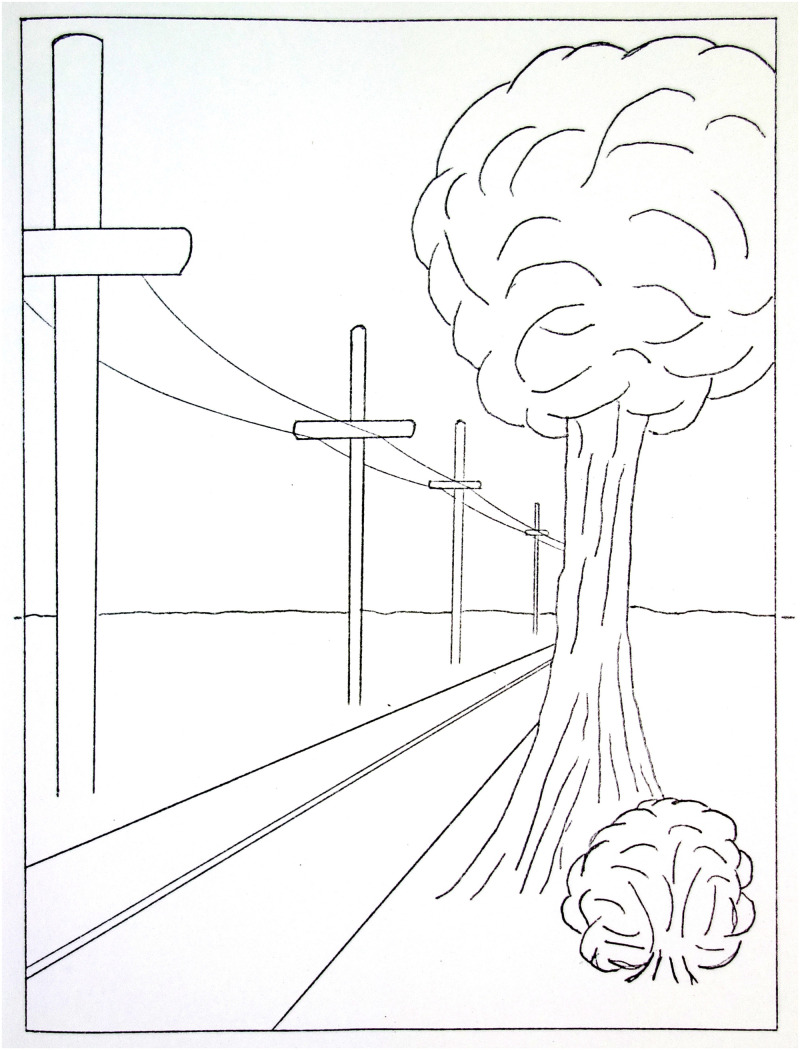
The horizon establishes a scale of height for all objects on the ground. (From Sedgwick, 1973.)

Assuming the horizon of the ground plane to be at a very large, effectively infinite, distance, then the line of regard to the horizon is effectively parallel to the ground. The perpendicular distance between the ground and the line of regard to the horizon is thus always the same and is always equal to the height of the eye above the ground. This establishes a scale factor across the entire ground. For any object resting on the ground, the portion of the object below the horizon is equal to the eye height (Figures 7 and 8).

**Figure 9.**
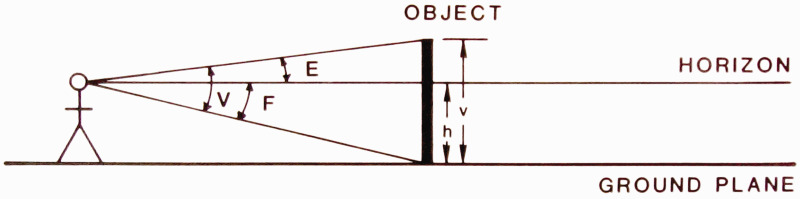
Horizon-ratio relation. The height (v) of every object standing on the ground surface relative to the height (h) of the point of observation is specified by the optic array angles E and F: v/h = (tanE+tanF)/tanF. When E and F are fairly small, this relation is closely approximated by the simple horizon-ratio relation: v/h ≈ V/F. (Adapted from Sedgwick, 1983.)

As shown in the Figure 9, there is a trigonometric relation here, such that total height of the object, relative to the eye height, is specified by the ratio of the tangent of the total visual angle subtended by the object to the tangent of the visual angle between the base of the object and the horizon. For moderately large distances, the ratio of tangents is closely approximated by the ratio of the angles themselves. I call this the *horizon-ratio relation*. There is now considerable evidence that this relation can be effective perceptually (Dixon et al., 2000; Leyrer et al., 2015; Mark, 1987; Warren, 1984; Warren & Whang, 1987; Wraga, 1999a, 1999b).

Like texture scale, the scale specified by the horizon-ratio relation does not depend at all on perceived distance from the observer.

**Figure 10.**
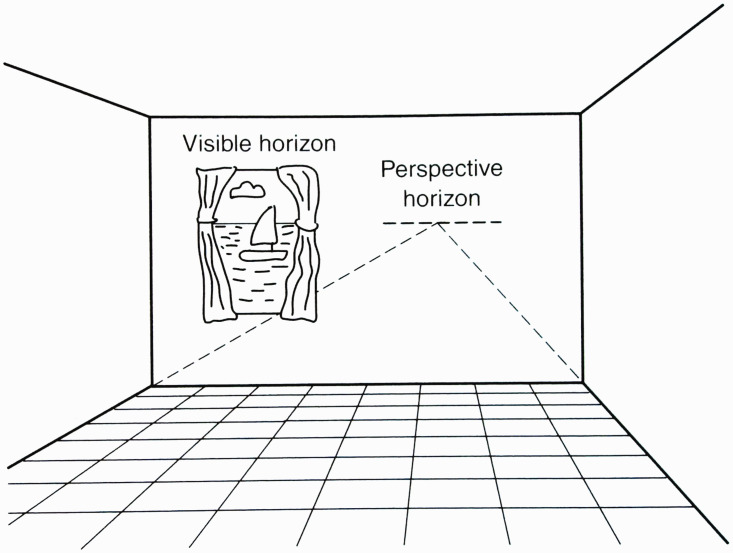
Horizon specified by linear perspective. On the left, the actual horizon is visible through the window. On the right, the horizon is hidden by the wall, but its location is revealed by the vanishing point of the room’s perspective. (Adapted from Sedgwick, 2010.)

*When the horizon is not visible*. Even when the horizon of the ground is not visible, the horizon-ratio relation can potentially be useful perceptually because the horizon’s location in the optic array can still be specified in several ways (Stoper & Cohen, 1991). The horizon is always located at eye level, which is moderately accurately perceived, even in the dark, based on vestibular and other proprioceptive information for the gravitational vertical. Additionally, all planar surfaces that are parallel to the ground share the same horizon; thus, its location can be found by extrapolating the projective convergence of such surfaces, such as the floor and ceiling of a room. Finally, the projections of parallel horizontal lines converge toward vanishing points on the horizon and so can specify its optic array location (Figure 10).

*Extended surfaces other than the ground plane*. In the terminology of linear perspective, the true horizon is the “vanishing line” of the ground plane. Every planar surface, of whatever orientation, has a vanishing line that it shares with all other planar surfaces that are parallel to it. The horizon-ratio relation can be generalized to all such surfaces, although then perhaps it should be referred to as the vanishing-line-ratio relation. For example, this generalized relation applies to perceiving the sizes of objects that are resting on an upward-slanting ground plane, or are suspended from a ceiling, or are attached to a vertical wall. The vanishing lines of non-horizontal planes, however, are not perpendicular to the direction of gravity and so lack that source of information to specify their location in the optic array. Non-horizontal planes are also less likely than the actual ground to extend so far from the observer that their boundary can function usefully as a visual approximation to their vanishing line. Finally, the perpendicular distance from the eye to a non-ground surface (i.e., the “eye-height”) may be less readily perceived.

*Horizon scales with motion parallax and binocular disparity*. Just as the line of regard to the horizon is parallel to the ground, the lines of regard from the two eyes to a location on the horizon are parallel to each other; likewise, the successive lines of regard from a laterally displacing viewpoint to a location on the horizon remain parallel to each other. These similarities imply that analogous geometrical relations involving horizon ratios exist for both binocular disparity and motion parallax, and in both cases, they establish a scale of size, relative to the observer, over the entire ground plane. The size of the separation between the two eyes, or between the successive positions of a moving eye, establish scale factors analogous to the scale factor established by the eye-height of the observer in the horizon-ratio relation (Sedgwick, 1973, Figure 11).

As with the ground-plane horizon-ratio relation, the scale factors established by motion parallax and binocular disparity both function independently of distance; they both can be generalized to the vanishing lines of other planar surfaces; and they both apply simultaneously to every object in the scene.

**Figure 11.**
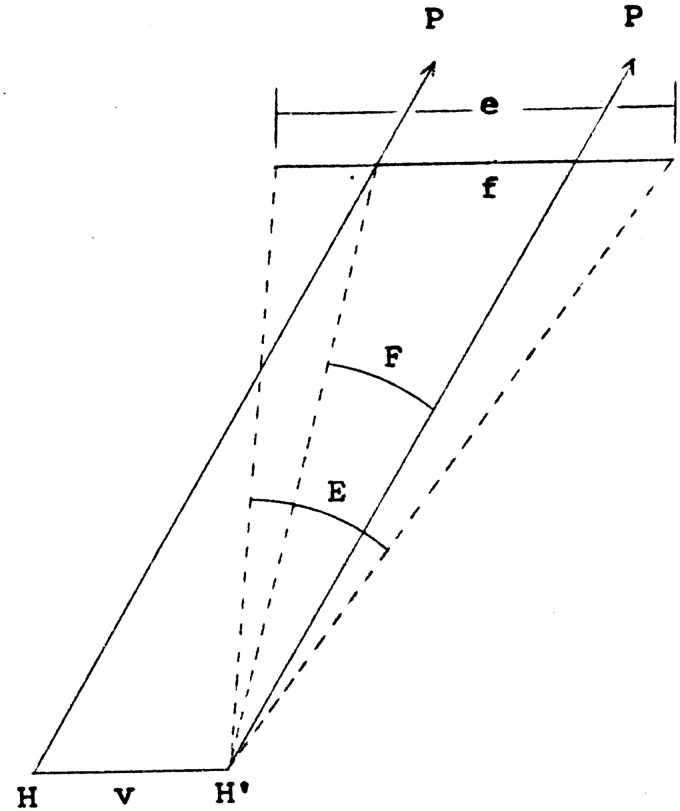
Horizon-ratio scale from motion parallax. In this diagram, the line e represents an extent in the environment. The point of observation moves parallel to e, from H to H’, through an extent v. Successive lines of regard to a point P on the horizon are parallel to each other, thus establishing at every distance a scale factor equal to v; thus, the extent f is equal to v. If the visual angles E and F are reasonably small, then e/v ≈E/F. (From Sedgwick, 1973.)

### Linear-Perspective Scale

Like the horizon-ratio scale, linear-perspective scale depends upon parallel lines being everywhere equidistant from each other. A pair of such lines determine a plane and establish a constant scale factor within that plane. The relative extents of edges lying in that plane can potentially be determined by their ratio relations with this constant scale factor (Sedgwick, 2001b).

**Figure 12.**
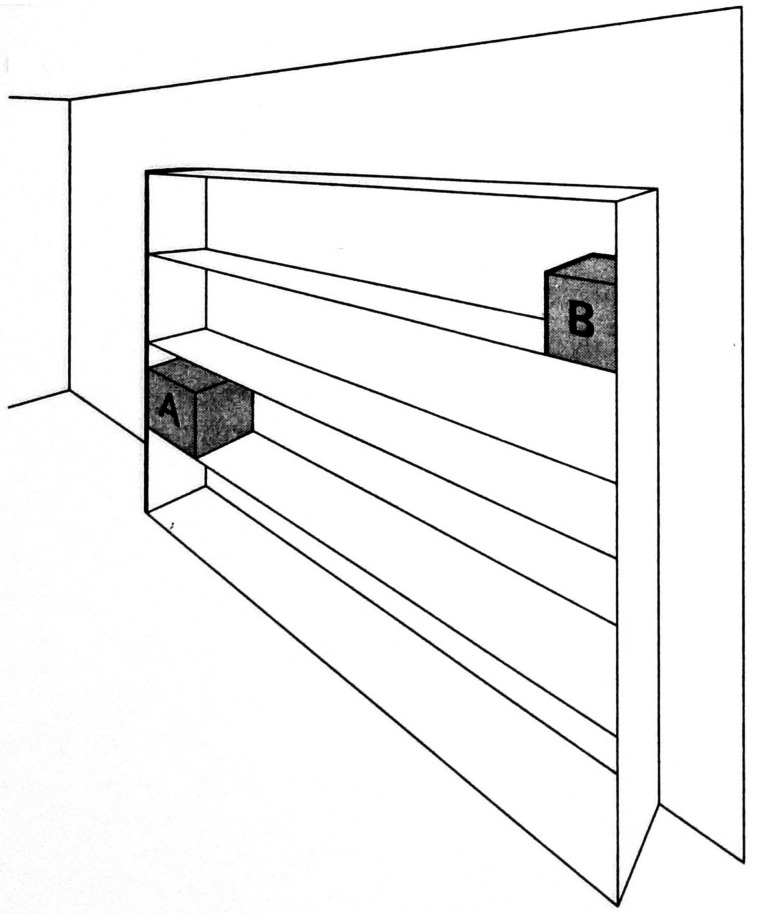
Linear perspective scale. (Adapted from Sedgwick, 2001b.)

In the example illustrated in Figure 12, the shelves of the bookcase establish a scale by means of which the physical sizes of the two boxes, A and B, can be compared (i.e., it can be seen that A is physically larger than B, although farther away and hence subtending a smaller visual angle).

**Figure 13.**
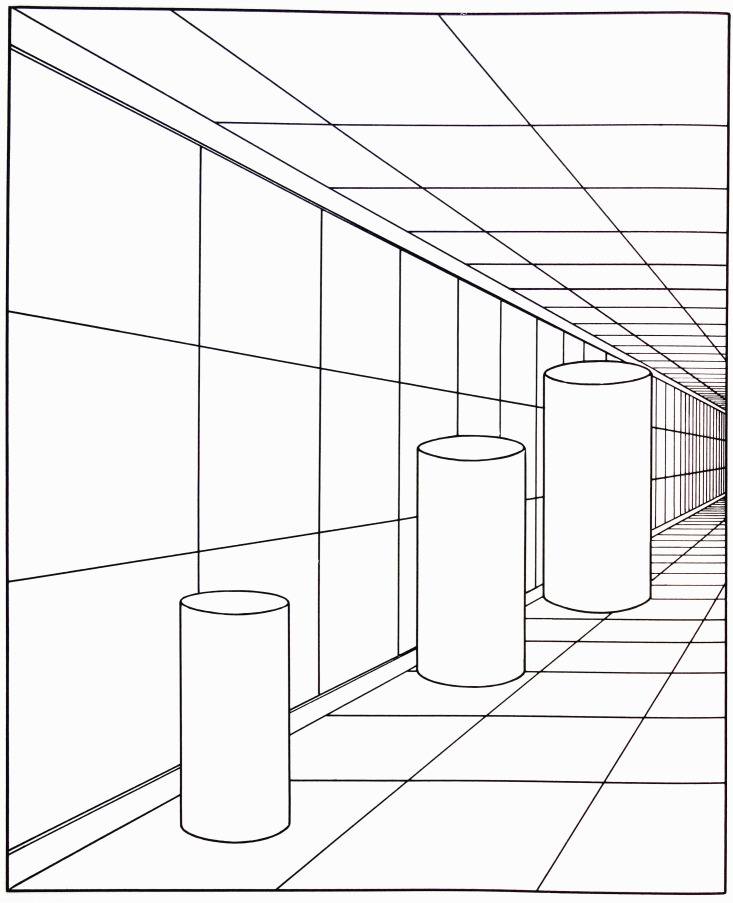
“Size as determined by distance.” This caption reflects Gibson’s theoretical position at the time. (Adapted from Gibson, 1950a.)

Examples of linear-perspective scale, such as the bookcase in Figure 11, are very common in carpentered environments. In addition, some powerful visual illusions, although often attributed to illusions of distance, also include linear perspective scale. One example is Gibson’s corridor illusion from the *Visual World* (Figure 13).

As Gibson points out, viewed as a representation of a 3D scene, there is no illusion here: Three cylinders of increasing size are shown lined up along the wall of a corridor. It is only if one attempts to ignore the 3D representation and estimate the size of the cylindrical shapes on the flat page that one still irrepressibly sees the cylinder on the right as larger and the cylinder on the left as smaller, even though they are all exactly the same size on the page. The strength of this illusion is of interest here for showing the powerful perceptual effect of the 3D representation.

This figure is a good example of linear-perspective scale because the relative height of each cylinder, within the 3D scene, is specified by the ratio of its height to the adjacent vertical separation between the two horizontal lines representing the bottom edge and the top edge of the wall of the corridor. There is also texture-scale information in this figure: The relative widths of the cylinders are specified by the ratios between the width of the bottom edge of each cylinder and the width of the floor tile that it covers. Finally, there is also horizon-ratio scale information in this representation. Although the horizon of the floor plane is not visible, its location in the scene is specified clearly by the many horizontal lines of the floor, wall, and ceiling—all converging toward a vanishing point on the floor’s horizon. That all three kinds of scale are present here is likely to be more the norm than the exception in carpentered environments.

What is most striking here, to this reader at least, is the caption of Gibson’s figure: “Size as Determined by Distance.” For each of the three scales discussed above, perceived size depends on a local ratio between some aspect of the object and some aspect of its context; perceived distance is in no way involved in any of the three.

Distance information in the picture is limited. The frame of the picture cuts off the ground just in front of the near cylinder; thus, the ground texture between the observer and the near cylinder is largely hidden, making this source of information for distance unavailable. The picture does include distance information, however, based on the varying elevation of the cylinders in the visual field, and this may also contribute to the perceived relative sizes of the cylinders.^11^ Experimentation with variants of Gibson's corridor figure could attempt to tease out the relative effects of elevation and of the three kinds of scale, but it is plausible to hypothesize that they all contribute in some degree to the strength of the illusion.

Gibson's caption of the figure shows the extent to which, in 1950, he was still *assuming* that only accurately perceived distance could determine accurately perceived size. By 1959, as we have seen, Gibson’s concept of scale had been more fully worked out, so that this Cartesian assumption could be abandoned.

### Absolute and Relative Scale

Absolute scale refers to a scale factor that is specified relative to the observer’s body. In most common circumstances, an observer may be presumed to be aware of their eyes’ current height above the ground. Thus, in such circumstances, the horizon-ratio scale allows the vertical extent of other objects on the ground to be compared directly to the size of the observer’s own body. In some circumstances, however, the observer may be resting on one surface but looking at objects resting on another surface whose height relative to the observer is not perceptible. This may occur, for example, in looking out the window of a building or airplane. An equivalent example occurs in looking at a photograph in which the height of the camera above the ground is not perceptually available. In these circumstances, absolute scale is not available from the horizon-ratio scale. Analogously, the other sources of information for scale can normally be related to the observer, but in some special situations they cannot be.

With respect to horizon-ratio scale, even if the observer’s eye-height is not perceptually available, the horizon still determines a relative scale for all objects resting on the ground. Thus, the vertical extents of all those objects relative to each other are still visible even though their vertical extents relative to the observer are not.^12^ With the other sources of scale information discussed above, there are again analogous forms of potential relative scale information available in viewing situations that do not allow for the perception of absolute scale.

### Context

An object’s relation to the texture of the ground, to the horizon, and to parallel lines of linear perspective (if present) are all, more broadly speaking, examples of the object’s relations to the environmental context that surrounds it. These examples are distinctive in that each is amenable to clear specification, but they do not exhaust the ways in which an object can be perceptually related to its context. In 1959, Irving Rock and Sheldon Ebenholtz published an experimental paper titled “The Relational Determination of Perceived Size.” Like Gibson’s 1959 paper, their paper attacked the Cartesian premise that perceived size depends on perceived distance, but they were most strongly motivated by research within Gestalt psychology showing the effects of frames of reference in areas such as the perception of velocity (Brown, 1931) and the perception of achromatic colors (Wallach, 1948).

Using highly simplified displays, presented in the dark, of two luminous lines, each surrounded by a luminous rectangle, Rock and Ebenholtz showed that observers' perception of the relative lengths of the two lines was strongly affected by the relative sizes of the two rectangles. Even though observers saw the sizes and distance of the rectangles approximately correctly, their perceptual responses were most strongly influenced by the contextual relation between the lines and their surrounding rectangles. Observers’ size perception was also influenced by the perceived distance of the lines, but more weakly, on average. This experiment suggests that in a normally complex environment, there could be many diverse frameworks that could contribute to the perceived scale at various places in the scene (see also Bennett & Warren, 2002).

There is substantial experimental evidence examining the strength of the linkage between perceived distance and perceived size; this evidence has been reviewed multiple times (e.g., Epstein et al., 1961; Gillam, 1995; Kilpatrick & Ittelson, 1953; Sedgwick, 1986). The clear conclusion is that although there is typically some effect of perceived distance on perceived size (or vice versa), the effect is variable and often fairly weak. This conclusion is far removed from the Cartesian assumption that the perception of size depends entirely on the perception of distance.

### Potential and Effective Information for Distance Along the Ground

Apart from the question of its role in the perception of size, distance has its own part to play in perception. As has been discussed above, Gibson’s ground theory substituted distance along the ground surface, and other extended surfaces, for the Cartesian theory’s distance through empty space; he then reformulated the traditional list of “cues” for distance into various kinds of gradients over the ground (Gibson, 1947, 1950a). The emphasis in his discussions of distance was generally on relatively large, outdoor spaces. Gibson himself did not have a program of research on distance perception. He did, however, carry out the first outdoor experiment, using large distances, on size-at-a-distance, as discussed above. His finding that size constancy did not diminish even at great distances, although variability increased, has led to a substantial number of experiments that did measure perceived distance, using various methods, over large outdoor distances along the ground.

In Gibson’s review, in *Ecological Approach*, of research related to his theory of layout perception, the only distance perception study he discusses is by J. Purdy and E. J. Gibson (Gibson, 1979, p. 161; Purdy & Gibson, 1955). In that experiment, the observer instructed a researcher to adjust a marker so that it bisected a stretch of distance between the observer and a farther marker. The total distance was as great as 275 yards (251.5 m), yet in spite of the much greater projective compression of the farther stretch of ground, the bisection settings showed no perceptual compression of the farther relative to the nearer distance. Later research using variants of this method have largely substantiated these results (Cook, 1978; Da Silva, 1985). Research using other methods has also shown, for the most part, reasonably linear distance perception, on average, out to substantial distances, although commonly with large individual differences ( for reviews, see Brenner & Smeets, 2018; Cutting & Vishton, 1995; Da Silva, 1985; Daum & Hecht, 2009; Durgin, 2014; Foley et al., 2004; Gillam, 1995; Howard & Rogers, 2008; Li et al., 2011; Sedgwick, 1986; Wagner, 2006). The largest distances investigated are reported in an experiment in which observers standing on a small island made absolute distance estimations over the sea to other islands and artificial objects out to distances of 15 km; their results followed a power function with an exponent of unity (Higashiyama & Shimono, 1994).

Somewhat different results have been obtained in experiments mapping out perceived distances among a group of markers distributed over a delimited region of an extended ground terrain. The general result has been that the depth component of the obtained map is somewhat compressed relative to the frontal component; the observed compression varies from 15% to about 50% (Levin & Haber, 1993; Loomis et al., 1992; Toye, 1986; Wagner, 1985, 2006). Loomis (2014) reviews theories that attempt to account for the difference between these results and those for perceived distance from the observer.

Much of the potential information for distance is difficult to manipulate in large outdoor spaces. As a result, most of the experiments done over large distances have not attempted to do so. Thus, their results describe the achievements of natural outdoor distance perception without attempting to experimentally determine the effective information. Gibson, however, hypothesizes that the good performance in the experiment by J. Purdy and E. J. Gibson (1955) is due to detecting and equating the *amount of texture* (e.g., number of clumps of grass) between the markers in the nearer and farther distances; he describes this as in *invariant* because the amount of texture included in a given extent is the same at varying distances from the observer and is also the same for frontal extents and extents in depth (Gibson, 1979, pp. 161–162). “Amount of texture” evidently is replacing the gradient of texture density as Gibson’s hypothesized effective information for distance perception. Using a different term with the same meaning, Gibson here closely follows his earlier discussion of “texture numerosity” (Gibson, 1959).

A number of experiments have been done in more controlled indoor situations to tease out the relative effectiveness of different kinds of texture information for distance ( for reviews and analysis, see Brenner & Smeets, 2018; Cutting & Vishton, 1995; Gillam, 1995; Howard & Rogers, 2008; Sedgwick, 1986). Among the results are that surfaces with texture tend to be more effective than surfaces without texture; regular textures tend to be more effective than irregular textures; projective texture shear tends to be more effective than projective texture compression, and texture elements that have an appreciable size tend to be more effective than texture elements that are dots.

The gradients of motion parallax and of binocular disparity, which featured prominently in *Visual World*, are not mentioned in Gibson’s discussion of the J. Purdy and E. J. Gibson experiment. Although observers had binocular vision and unconstrained head movements, perhaps Gibson regarded these sources of potential information as ineffectual for the bisection of the large distances of this experiment (Gibson, 1979, pp. 161–162). These two sources of information are not totally ineffectual, however, at such large distances. In research done in total darkness in an abandoned railway tunnel, with the “near” LED at 40 m from the observer and the far LED at depths ranging from 0 to 248 m from the near LED, it was found that “binocular, but not monocular, estimates of the depth between pairs of LEDs increased with their physical depths up to the maximum depth separation tested” (Palmisano et al., 2010). Dynamic viewing was also tested, with the observer moving their head laterally by up to 1.5 times the interocular distance. The researchers report that “[d]ynamic binocular viewing was found to produce the greatest (i.e., most veridical) estimates of depth magnitude, followed next by static binocular viewing, and then by dynamic monocular viewing. (No significant depth was seen with static monocular viewing.)” (Gillam, Palmisano, et al., 2011). Finally, using two observation distances (20 and 40 m), they found that depth estimates were scaled for the observation distance when the interior of the tunnel was illuminated up to the nearest LED. Thus, both motion parallax and binocular disparity can provide some useful depth information at distances up to almost 300 m.

In *Visual World*, Gibson lists “Relative upward location in the visual field” as a possible “clue” to an object’s distance, both in the world and in pictures. Gibson describes this clue as “an inference, or a probable indicator of distance, not as a true stimulus.” He makes this distinction, at least in part, because this clue is only valid when the object is in contact with the terrain (Gibson, 1950, pp. 141, 180). He does not mention this clue at all in *Ecological Approach*, suggesting that he did not regard it as potential information for distance.

Although, I have suggested above, Gibson implicitly made use of ecological constraints in considering potential sources of visual information for perceiving the layout of surfaces, he did not, to my knowledge, explicitly formulate or explore the concept of such constraints. It seems, however, from the example of “upward location” that he might not have accepted that potential information can be contingent on specified constraints that may or may not be not be satisfied in natural environments. For example, in a typical crowded layout, many of the objects are not in contact with the ground (or floor); they are resting on tables or shelves, attached to a wall, or hanging from the ceiling; their distance is not related to their upward location in the visual field. Yet, as is discussed in the following section of this paper, there is potential information in the optic array to specify whether or not an object is in contact with a surface such as the ground. *If* such contact exists, *then* the angle of elevation in the optic array of the object’s ground contact location *specifies* its distance along the ground from the observer; this distance is scaled, in a simple geometrical relation, by the eye-height of the observer. That observers are able to make effective use of such contingent information has been shown experimentally. Using pictorial displays, the researchers found that increasing elevation in the field led to increasing perceived distance *if* the object was perceived to be in contact with the floor; but *not if* it was perceived to be in contact with the ceiling (Gardner et al., 2010).^13^ This implies that forms of potential information can interact, with one form of information (e.g., contact) satisfying the conditions necessary to validate another form of information (e.g., elevation in the array).

## Orientation

### Slant in the Perception of the Visual World

In the Cartesian theory, as noted above, the perception of the 3D world is based on the perception of the direction and distance of points. The shape and slant of a surface are thus reduced to the varying directions and distances from the observer of points on that surface. In contrast, in Gibson’s ground theory, distance from the observer does not enter into the perception of surface slant. In *Visual World*, Gibson hypothesizes that “when a three-dimensional physical world is projected optically, the slant and shape of its surfaces undergo a mathematical transformation in the projection,” such that “the surfaces, slopes, and edges of the world have correlates in the retinal image specifically related to their objective counterparts by a lawful transformation” (p. 9). For example, if a slanted surface is textured, then in the retinal image there is a gradient of texture density that “may decrease upwards, from left to right, right to left, or downwards, and these are the four respective conditions for a floor, a left-hand wall, a right-hand wall, and a ceiling” (pp. 70–71). The perceived slant of the surface is hypothesized to be in direct psychophysical correspondence with the gradient of texture in the retinal image.

**Figure 14.**
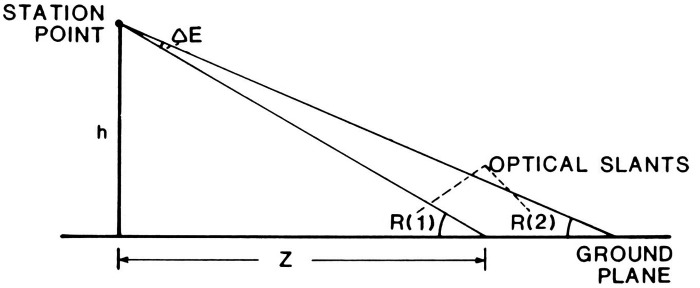
Optical Slant. R(1) = arctan(h/Z). This relation generalizes to planar surfaces of any geographical slant. (Adapted from Sedgwick, 1980.)

Gibson’s treatment of slant is a radical departure from the Cartesian theory. Not surprisingly, then, in *Visual World*, this conceptually new approach is not yet fully developed. The concept of slant is inherently relational; it is the orientation of a surface relative to some reference. In the *Visual World*, Gibson does not yet distinguish between two definitions of slant, which were later labeled “geographical slant” and “optical slant” (Gibson & Cornsweet, 1952). “Geographical slant” is slant defined with reference to the horizontal ground plane of the environment. “Optical slant” is slant defined with reference to the viewer’s line of regard (Figure 14).

Fundamental differences exist between these two kinds of slant. The geographical slant of a plane surface is the same everywhere on the surface; for example, if the surface has a geographical slant of 45°, then every patch of that surface is slanted by 45° from the horizontal. In contrast, a plane surface’s optical slant changes continuously as a viewer’s line of regard sweeps along a surface, in a straight-line path, in any direction. This is perhaps most evident with respect to the horizontal ground plane, extending toward infinity. The line of regard to the location directly beneath the observer is perpendicular to the ground, but the line of regard to the horizon is parallel to the ground; as the line of regard scans along the ground from near to far, the optical slant of each successive location changes progressively from the first to the second of these two extremes. Throughout the *Visual World*, Gibson simply uses the term “slant,” not yet having distinguished between geographical and optical slant. Based on his usage of the term, however, it seems as though what he has in mind is a mixture of the two meanings of the term.

### The Emergence of Optical Slant and Geographical Slant

In 1950, Gibson published a paper on the perception of visual surfaces (Gibson, 1950b). The paper included an experiment showing that the perceived slant of a surface varied in the predicted direction as the density of its projected texture varied. Because he had not yet distinguished between optical and geographical slant, however, the experiment was set up in such a way that those two types of slant were confounded.

Gibson must have been made aware of this problem not long after *Visual World* and the paper on visual surfaces were published. In 1952, he and Janet Cornsweet published a paper in which they named optical and geographical slant, made explicit the distinction between them, and acknowledged the previous failure to note this distinction (Gibson & Cornsweet, 1952). An experiment in this new paper presented participants with two different displays and tasks, one requiring a response in terms of optical slant and the other a response in terms of geographical slant. The experiment is somewhat weak methodologically,^14^ but its results do suggest that observers were able to give perceptual responses based on either optical slant or geographical slant, as required, and hence that optical slant is accessible to perceptual report. Concerning optical slant, it concludes that “[t]he hypothesis that this quality corresponds to the gradient of density of ‘texture’ at the fovea becomes reasonably certain.”

Because Gibson’s hypothesis of psychophysical correspondence now becomes associated with the concept of *optical* slant, it is of interest to see how Gibson and Cornsweet deal with their concept of *geographical* slant. Here they give an extended description of a situation in which the differences between the two concepts are most distinct to them:

Take as an example the visual experience of a man standing on a level desert plain and looking about … .What he sees is a level ground extending to the horizon with himself standing on it. No impression of slant seems to be evident. Ordinarily the man is unaware of his saccadic eye-movements, but if he attempts to introspect, he may discover that every fixation yields a clear momentary impression of a small segment of the ground which *does* have a kind of slant. As he looks downward toward his feet the slant approaches zero, as he looks upward the slant increases, as the center of clear vision approaches the horizon the slant becomes maximal, and at the horizon itself the land ceases to be a surface and becomes an edge

In this situation the momentary impressions of slant quickly add up to the experience of a single surface perpendicular to gravity … .In this situation the total perception is a product not only of successive retinal images but almost certainly of correlated postural gravitational stimuli as well. Finally, in this situation the optical slant of the surface at the point of regard is not congruent with the geographical slant of the surface in the visual world. (p. 11)

It is clear here that it is geographical slant that is being associated with the visual world. The description of optical slant sounds more like the visual field:^15^ a momentary impression that requires introspection to be noticed. What is also of interest is that geographical slant is postulated to be obtained from optical slant by quickly adding up the momentary impressions obtained from a series of fixations, correlated with postural gravitational stimuli.

### Deriving Geographical Slant From Optical Slant

**Figure 15.**
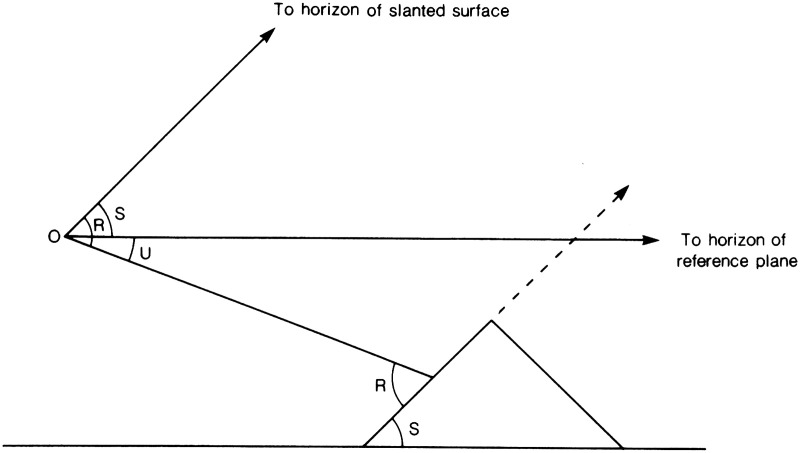
The relation between optical slant and geographical slant. (Adapted from Sedgwick, 1986.)

An important clarification of the relation between optical and geographical slant is presented in the dissertation (also written up as a technical report) of Gibson’s student W. C. Purdy (Purdy, 1960), which also contains the first mathematical analysis of texture gradients. Purdy’s analysis is in terms of the newer concept of the optic array (discussed above) rather than the retinal image. Using “line of regard” to refer to an angular direction in the optic array rather than to an angular rotation of the eye, Purdy defines optical slant as the angle at which the line of regard meets a point on a surface. He then describes a simple relation between the optical slant and the geographical slant of a plane surface (p. 18). Putting Purdy’s words into a simple equation, if we let R be the optical slant of a location on the surface, let U be the angle of declination of that location from the horizontal, and let S be the geographical slant of the surface, then S = R – U (see Figure 15). This relationship is simpler than the one hypothesized by Gibson and Cornsweet, both because it does not involve eye position information and also because it does not involve scanning the surface and adding up successive optical slants; any one optical slant is sufficient to specify the surface’s geographical slant relative to a horizontal reference.

Purdy assumes, as do Gibson and Cornsweet, that geographical slant is not available perceptually unless it is derived from optical slant; thus, he does not mention that his equation would also allow optical slant to be derived from geographical slant, if the latter were independently available perceptually.

### Vanishing Points and the “Ghost Image”

**Figure 16.**
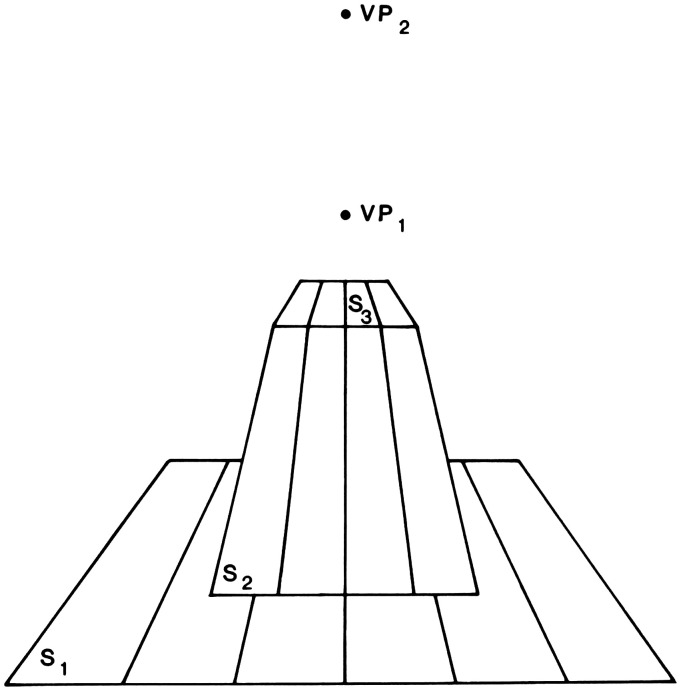
Observer’s view of a 3D surface layout. (Adapted from Sedgwick, 1983, after Hay, 1974.)

A critical development, in my view, was produced by John Hay, another student of Gibson’s (Hay, 1974). Hay’s analysis concerns geographical slant, but instead of using that term, he refers to an “environmental layout” of surfaces. Importantly, Hay does not derive geographical slant from optical slant; instead, he bases his analysis on linear perspective and uses vanishing points to specify this environmental layout directly. Hay’s argument is best understood by reference to two of his figures. One figure (Figure 16) shows the picture plane projection of an observer’s view of a 3D surface layout: a horizontal floor plane (S1) and a planar ramp that is slanted upward (S2) to join a plane that is parallel to the floor (S3). The surfaces all appear to be rectangular, with the projections of their parallel sides converging toward their vanishing points, which are also shown. The sides of S1 and S3 are all parallel to each other in the environmental layout and so they share the same vanishing point (VP1) on the projection plane. The projections of the parallel sides of S2 converge toward a vanishing point (VP2) that is higher in the projection plane than VP1.

**Figure 17.**
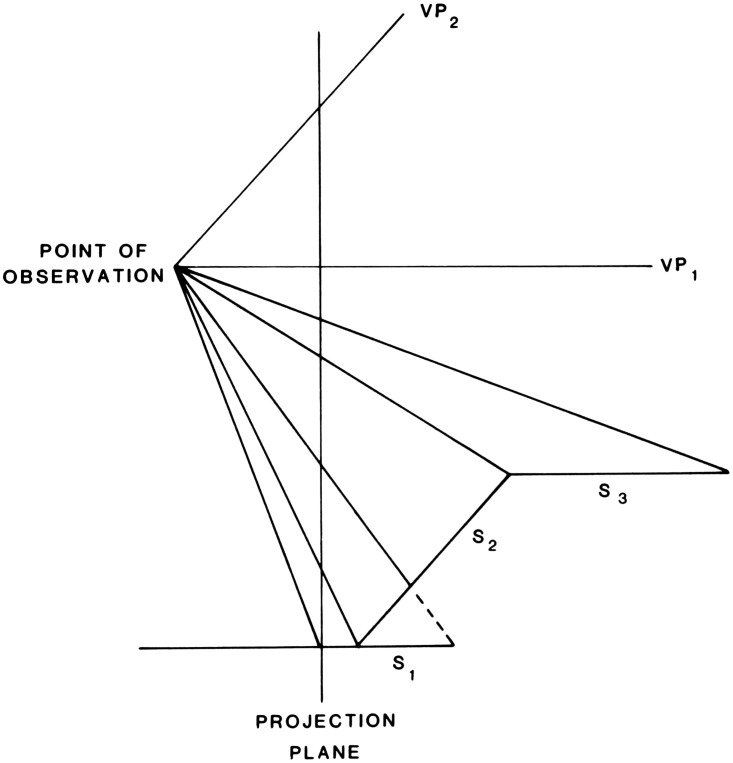
Side view of a 3D surface layout. (Adapted from Sedgwick, 1983, after Hay, 1974.)

Hay’s other figure (Figure 17) shows a schematic side view of the observer, the picture plane, and the three planar surfaces, with S1 and S3 appearing as horizontal lines, and S2 appearing as a slanted line. Also drawn are what Hay calls the “vanishing rays,” which are the optic array lines of regard parallel to each surface; each vanishing ray of a surface is headed toward the vanishing point that Hay associates with that surface. There are only two vanishing rays: the ray headed toward VP1 is parallel to both S1 and S3, and the ray headed toward VP2 is parallel to S2; Hay refers to these two vanishing rays as VR1 and VR2, respectively.

Once these figures are understood, Hay’s conclusion is clear: “the angle between the vanishing rays VR1 and VR2, in Figure 17 must be exactly equal to the angle between the environmental surfaces S1 and S2.” That is, Hay is saying that the environmental orientation of S2, relative to the horizontal floor, S1, must be equal to the angle between the vanishing rays, VR1 and VR2, of these two surfaces, because each vanishing ray is parallel to its respective surface.

The angle between VR1 and VR2, seen from the side, seems readily apparent to someone viewing Figure 17; this, however, is not the view of the observer. In the view of the observer, shown in Figure 16, it is the angular difference in direction of the two vanishing points, VP1 and VP2, that specifies the environmental orientation of S2 relative to the horizontal reference surface, S1.

The relationship described by Hays is both elegant and simple. It is theoretically important because *geographical slant is not derived from optical slant*, but instead is obtained directly from the relation between vanishing points in the optic array.

Hay’s analysis postulates a one-to-one relationship between the locations of vanishing points in the optic array and orientations of surfaces in the environment. What Hay is postulating, however, is something more than a relationship of *correspondence*, in Gibson’s sense; it is a relationship of *identity*. That is, for Hay, the orientation of the vanishing line of a surface *is* the orientation of the surface; they are parallel and so have the same orientation. Thus, for Hay, the pattern of vanishing points produced by a layout of environmental surfaces replicates the orientation structure of that layout. He calls this pattern of vanishing points the “ghost image.” Moreover, because the vanishing points are located at infinity, their locations in the optic array are unchanged by movements of the point of observation; Like the stable environmental layout, the ghost image is unaffected by observer motions.

In his paper, Hay discusses how the ghost image can be used to analyze several perceptual problems, such as the question of how the virtual space of a picture is distorted when the observer is not viewing the picture from the correct viewpoint.^16^

What does not concern Hay is the question of how the observer picks up the optic array locations of the vanishing points, such as VP1 and VP2. These locations are conveniently marked and labelled in Figure 16, but not in the optic array of the observer. The secondary status of this question for Hay reflects the shift, discussed above, that occurred in Gibson’s theory around 1960–61, after the work of Purdy, but before that of Hay.^17^ In Hay’s formulation, the concept of psychophysical correspondence with properties of the retinal image is gone. It has been replaced by the concept of invariant relations between the optic array and the environment; this is no longer thought of as a stimulus to perception; instead, it is *potential* information that an observer may or may not be able to use.

Hay mentions three types of “higher-order variables” in the optic array that may help to locate the monocular vanishing points of a 3D layout: outline form (such as the converging forms in Figure 16), texture gradients (indicated in Figure 16 by the parallel lines on each surface), and motion perspective (Hay’s does not extend his analysis to binocular vision). But Hay does not attempt to analyze how this would happen, nor does he assert that these higher-order variables will always be sufficient to locate the vanishing points of an environmental layout of planar surfaces.

### The Invariant Perspective Structure of the Optic Array

**Figure 18.**
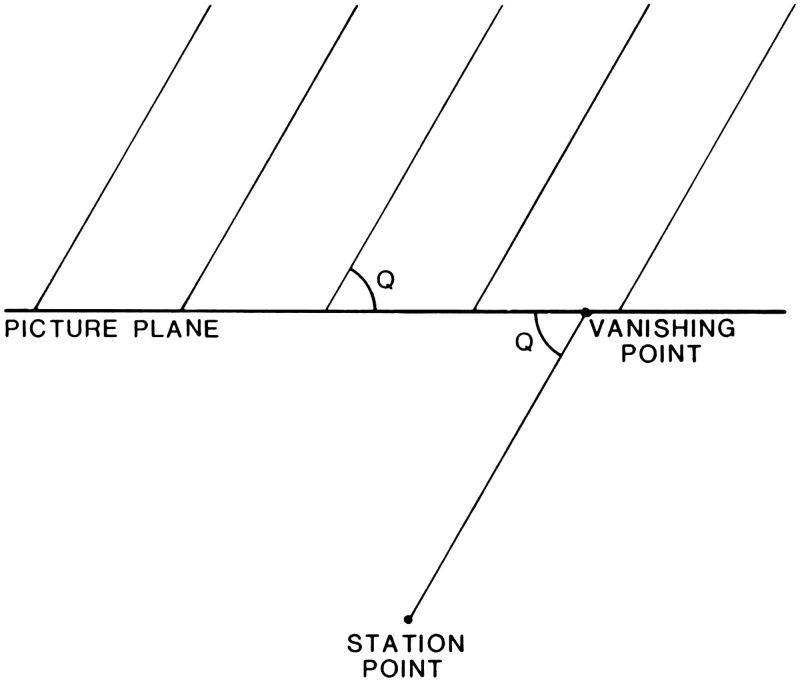
Parallel lines & their vanishing point. The orientation of the line of regard to a vanishing point is identical to the orientation of all lines sharing that vanishing point. (Adapted from Sedgwick, 1980.)

Although it does not affect his key insights, there is a significant misstep in Hay’s analysis. Hay’s Figures 16 and 17 are misleading, and it seems that Hay himself was misled by such figures. Hays analysis properly applies to the geographical orientation of *lines*, not of *planes*. Straight lines that are parallel to each other all have, by definition, the same geographical orientation. If a straight line belonging to that family of parallel lines passes through the point of observation, then that line is the vanishing ray of that family; their projections all converge toward a single point that lies on the vanishing ray and is their vanishing point. Thus, the direction of a vanishing point in the optic array specifies the orientation of *straight lines* that are parallel to the vanishing ray of that vanishing point (Figure 18).

**Figure 19.**
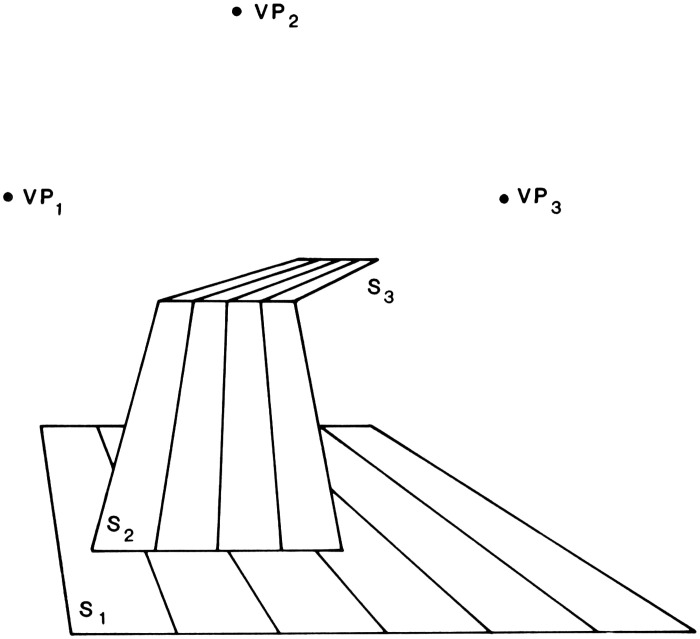
Surface orientation is not specified by a single vanishing point. (Adapted from Sedgwick, 1983.)

The projection of a planar surface does not have a *vanishing point* that specifies its geographical slant; it has a *vanishing line*. Without going into details, Hay’s figures only work because they embody additional, implicit constraints that allow one vanishing point to specify the orientation of a surface. In the general case, two vanishing points are necessary to specify a vanishing line, which in turn directly specifies the geographical orientation of a family of parallel planar surfaces. In Figure 19, I have modified Hay’s Figure 16 into a more general case that makes clear the error in Hay’s linking of vanishing points to geographical slant. In Figure 19, the two surfaces S1 and S3 have the same horizontal geographical slant as in Figure 16; but in Figure 19, the parallel lines forming the edges and in-lines of the surface S3 have a different orientation and hence a different vanishing point (VP3) than the edges and in-lines of surface S1, whose vanishing point is VP1.

Thus, Hay’s analysis is correct in its essential insight into the important potential role of perspective structure in the perception of 3D environmental layout, but his analysis needs to be extended to include the vanishing lines of surfaces. Such an analysis has been developed in some detail elsewhere (Sedgwick, 1983), but here only a brief overview is given.

**Figure 20.**
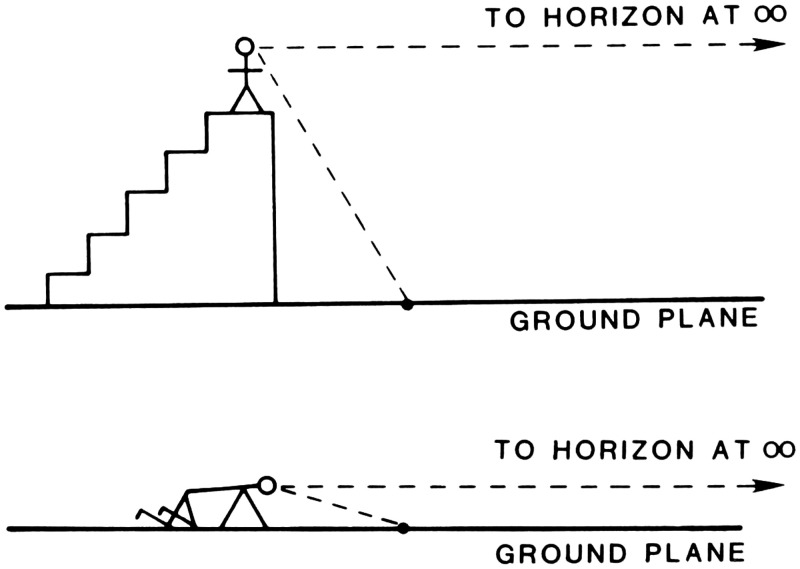
Optic array invariance of the horizon. (Adapted from Sedgwick, 1983.)

The 360° spherical optic array, centered on the viewpoint of the observer, is best suited for a description of the array of vanishing points and vanishing lines associated with an environmental layout of edges and surfaces. The horizon is the ground plane’s vanishing line in the optic array. It surrounds the observer and could be described as the horizontal great circle of the spherical optic array.^18^ The location of the horizon in the optic array is invariant; if the observer moves in any direction—up, down, forward, backward, right, or left—the location of the horizon in the observer’s optic array is unchanged (Figure 20).

Every plane that is parallel to the ground plane is also horizontal and has its vanishing line on the horizon. Thus, the horizon is not only the vanishing line of the ground plane; it is the vanishing line of the entire family of parallel horizontal planes. All of these horizontal planes have, by definition, the same geographical slant.

As we have already noted, even if the horizon is not visible, its optic array location is normally richly specified by the perspective convergence of horizontal environmental lines and edges, by vestibular input, and by many other perceptible effects of gravity. The horizontal is thus normally available perceptually as a reference in relation to which the geographical slant of other, non-horizontal surfaces may be perceived.

Consider, for example, a plane that is slanted up by an angle of 45° relative to the horizontal. Again, all planes parallel to this plane share the same vanishing line, including the parallel plane that passes through the point of observation. Thus, this vanishing line is another great circle of the optic array. The geographical slant of this plane, and thus of any plane in this family, is directly specified by the orientation of its vanishing line in the optic array (Figure 15). Generalizing, we can see that *there is a one-to-one relationship of identity between the geographical orientation of any family of parallel planar surfaces and the orientation of the vanishing line of that family in the optic array*.

Hay’s “ghost image” of vanishing points is another important source of information about 3D orientation, but as noted above, it applies to the geographical orientation of families of parallel *straight lines*, not planar surfaces. Thus, for an observer facing the rear wall of a rectangular room, the receding edges of the floor, walls, and ceiling are not all on the same plane, but these edges are all parallel to each other and their projections in the optic array converge toward a common vanishing point; the line of regard to that vanishing point is parallel to, and so directly specifies, the geographical orientation of all of those parallel edges.

I have referred to the combined pattern of vanishing points and vanishing lines in the optic array projection of a 3D layout of planar surfaces and straight edges as the “perspective structure of the optic array” (Sedgwick, 1983), but it is more accurate to call it “the *invariant* perspective structure of the optic array,” because it consists of a set of optic array structures that remain invariant under motions of the point of observation.

If there are more or less flat surfaces and more or less straight edges in a 3D layout of surfaces, then they will have geometrically determined vanishing lines and vanishing points in the optic array. These invariant perspective structures are not themselves visible lines or points in the optic array because they are projective limits that would only be visible if the surfaces or edges actually extended to infinity. Except under highly reductive conditions, however, there will be *potential* information that specifies the location in the optic array of these vanishing lines and vanishing points. For example, if a flat surface is textured then the limit of its optic array texture gradient specifies its vanishing line; if the observer is moving or is binocular, then the gradients of motion perspective or binocular array disparity also specify the surface’s vanishing line. Analogous potential information specifies the vanishing points of straight edges. Whether such *potential* information is *effective* information for a particular observer will depend upon that observer’s perceptual capabilities.

Earlier, we looked at Purdy’s demonstration (Figure 15), that a planar surface’s geographical slant can be derived from the optical slant of location on that surface, combined with a horizontal reference. Now, having seen that such a surface’s geographical orientation is directly specified by its vanishing line in the optic array, we can see that there is an invariant reciprocal relation between geographical and optical slant. As can be seen in Figure 15, *the optical slant (R) of a location on the surface is equal to the location’s angle of declination (R) below the vanishing line of the surface.* This *slant invariant* is *potential* stimulus information. The invariant relation between optical and geographical slant suggests that these two concepts of slant go together, rather than being in tension with each other. That is, in the act of looking at a surface location, both optical slant and geographical slant may be perceived simultaneously, each affording different but related aspects of behavior.

**Figure 21.**
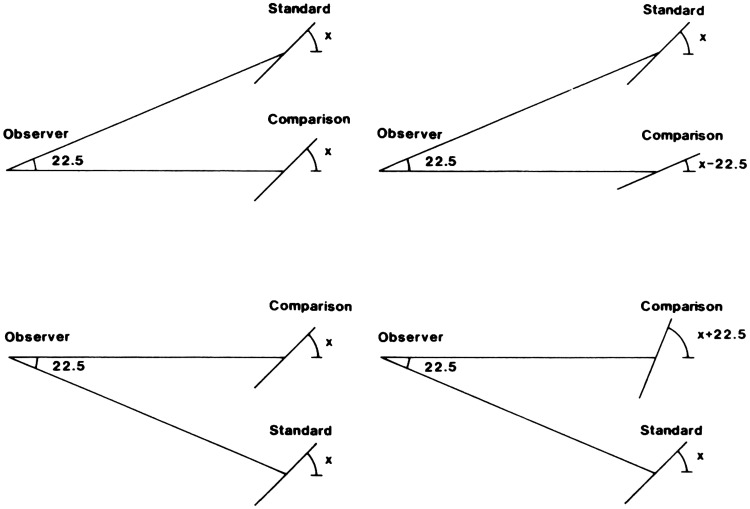
Geographical slant & optical slant matches. On the left observers match geographical slant by setting the comparison so that it looks parallel to the standard. On the right observers match optical slant by setting the angle of regard to the comparison to match the angle of regard to the standard. (Adapted from Sedgwick & Levy, 1985.)

Steven Levy and I carried out an experiment using computer graphics in which a variously slanted standard surface patch was projected 22.5° above or below a comparison surface patch; observers were asked to either (a) make the two surfaces parallel (i.e., equal geographical slant) or (b) equate the angle of each surface to the line of regard (i.e., equal optical slant; Figure 21). In our study performance was better for geographical than for optical slant instructions (Sedgwick & Levy, 1985); we interpreted this result as supporting the hypothesis that observers are able to perceive geographical slant directly rather than being obliged to derive it from optical slant. But our results also supported the earlier results of Gibson and Cornsweet, insofar as they showed that observers could attend to either geographical or optical slant, in accordance with the instructions that they were given.

### Implicit Linear Perspective

Linear perspective and texture gradients are closely related. This is particularly clear when the surface texture is composed of equal-size rectangular elements, such as bricks or tiles. Such textures, which could also be described as examples of linear perspective, are easier to analyze geometrically than natural textures such as grass or pebbles (Figure 2), and so they have often been used to represent or analyze the stimulus information carried by the optic array projection of surface textures. For example, Purdy (1960) used a grid of orthogonal straight lines to represent texture in his early analyses of texture gradients, and such grids have also commonly been used in representations of texture scale (Figure 6). Conversely, even without containing parallel lines, a texture of uniformly sized texture elements contains implicit linear perspective (Sedgwick, 1983). Figure 3, from Gibson (1950a), shows such a “texture” gradient composed of similarly sized objects (the tops of barrels).

**Figure 22.**
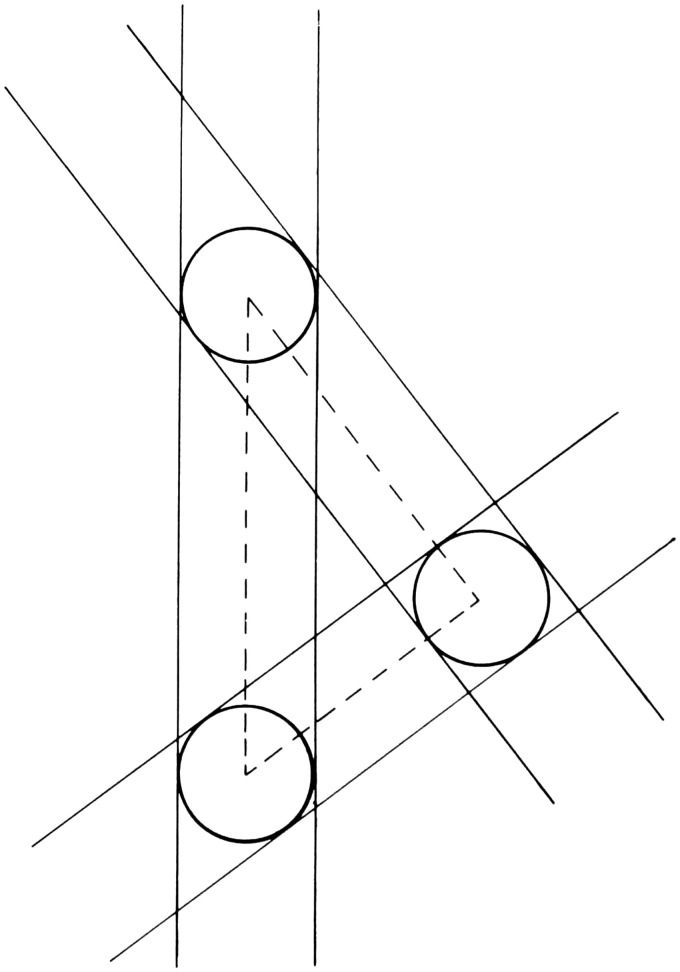
Parallel lines determined by circular texture elements. (Adapted from Sedgwick, 1983.)

**Figure 23.**
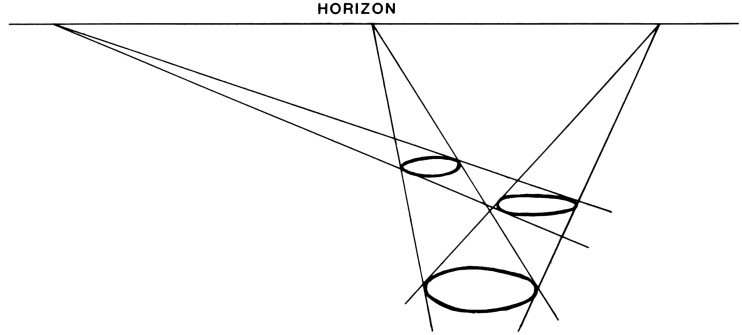
Horizon determined by circular texture elements. (Adapted from Sedgwick, 1983.)

Figure 22 shows that each pair of circular barrel tops implies a pair of parallel lines, tangent to the sides of the two circles. Each implicit pair of parallel lines converges in the optic array to a vanishing point lying on the vanishing line of the plane; taken together, these many vanishing points specify the vanishing line, and hence the geographical slant, of the plane, as shown in Figure 23.

If the texture elements are somewhat irregular in size or shape, this introduces noise into the determination of the vanishing line, with the extent of the irregularity determining the amount of noise (Sedgwick, 1983). Empirical studies of the perception of surface slant have generally found more accurate performance with regular textures than with irregular textures (Gillam, 1995).

In this section, we have examined the development of the concept of geographical slant, the demonstration of invariant structures in the optic array capable of directly specifying geographical slant, and the existence of some experimental data supporting the hypothesis that geographical slant is obtained directly from the optic array rather than being derived from optical slant. These developments undermine the reductive Cartesian assumption that the slant of a surface is based on the perception of the distance to the points comprising the surface, and they also question the more sophisticated but still reductive assumption of David Marr and many others that the geographical slant (or object-centered slant, as he called it) of an extended surface must be built up by putting together the optical slants, relative to line of regard, of many local surface patches.

### The Efficacy of Texture Gradients

Gibson himself abandoned the paradigm of studying the perceived slant of textured surfaces in isolation. The early research—his and that of others—produced results that were disappointing to Gibson, often finding that perceived slant was considerably underestimated in comparison to the geometrically predicted slant. In support of his negative evaluation of the empirical results, Gibson cites Gibson (1950b), Gibson and Cornsweet (1952), Beck and Gibson (1955), and Bergman and Gibson (1959). He also cites Flock (1964, 1965) and Freeman (1965) as “describing the complexities of the results” (Gibson, 1979, pp. 165–166). In his *Ecological Approach*, looking back on his earlier work, Gibson wrote: “I tried to reformulate the list [of cues to depth] in 1950 as ‘gradients and steps of retinal stimulation … The hypothesis of gradients was a good beginning, but the reformulation failed” (Gibson, 1979, p. 149). Concerning his research on the perception of slant in particular, he wrote:

Phenomenal slant does not simply correspond to the gradient …  (Gibson, 1979, p. 149)

I had made the mistake of thinking that the experience of the layout of the environment could be compounded of all the optical slants of each piece of surface. I was thinking of slant as an absolute quality, whereas it is always relative. (Gibson, 1979, p. 166)^19^

Nevertheless, Gibson’s idea that perceived slant is based on projected gradients of texture has been hugely influential, inspiring or provoking literally hundreds of articles and presentations on the subject, continuing up to present. Just as there are model organisms in the study of biology, perceived slant from texture has become a kind of model problem used in the investigation of broader theories that in some cases stray far from Gibson’s own (e.g., Landy et al., 1995). Gibson called for mathematical analysis (Gibson, 1950, pp. 9, 11) and many such analyses have been produced, in some cases accompanied by empirical data concerning human vision, but in other cases aimed at computer vision. Some forms of texture gradients have been found to be substantially more effective in human perception of slant than others, and the effort to systematize and understand these results is ongoing (e.g., Braunstein & Payne, 1969; Chen & Saunders, 2020; Knill, 1998; Todd et al., 2005, 2007; Todd & Thaler, 2010).

## Contact

### What Gives Rise to the Perception of Contact?

Perceiving an object’s size, distance, or orientation based on its relation to the ground depends on perceiving the location where the object contacts the ground. Thus, for the ground theory, perceived contact is logically prior to the perception of other spatial relations such as size or distance. This leads to the question of what gives rise to the perception of contact.

Moreover, Gibson’s ground theory, as noted above, is not limited to the ground surface. Gibson’s more general concept of visual space is “as a continuous surface or an array of adjoining surfaces” (1950a), such as the floor, walls, and ceiling of a room. The perception of contact, that is, where one surface adjoins another, is essential in perceptually holding together this array of surfaces.

In the Cartesian theory of space perception, on the other hand, the perception of contact is not a special problem. Objects and surfaces are conceptually reduced to points in space, and each such point is perceptually localized in space by its angular direction and distance from the eye. Contact is logically encompassed within this formulation. Two points are perceived to be in contact if (and only if) they have the same perceived direction and distance. I do not know of any discussion, within the Cartesian tradition, of spatial contact as a distinct question for visual perception.^20^

With Gibson’s rejection of Cartesian theory in favor of the ground theory, however, the question of how contact is perceived became a central question. Gibson was clearly aware that this question needed to be addressed. Early in his discussion of “the stimulus variables for visual depth and distance,” he writes “Let us make two assumptions about the typical physical world of ground and objects and assert that, first, objects tend to be in contact with the ground instead of up in the air … ” (Gibson, 1950a, p. 77). Here, as we have already seen in other contexts, Gibson is clearly taking an ecological approach long before he formalized this concept, and he is describing what we might today call an ecological constraint.

Later on, in discussing how the distance of an object is seen, Gibson follows through by assuming a tendency of visual perception to make use of this ecological constraint:

. . . [H]ow is the distance of an object fixed on the background? Let us assume that an object is seen where its contour interrupts the background—at that distance and no other—except when depth-at-a-contour brings it forward in distance. This latter effect is produced mainly by a step in the rate of deformation or disparity at the contour. We are assuming that in the absence of what is called relative motion or stereoscopic depth a contour is seen on the background. (pp. 177–178)

### Gibson’s Optical Contact Illusion

**Figure 24.**
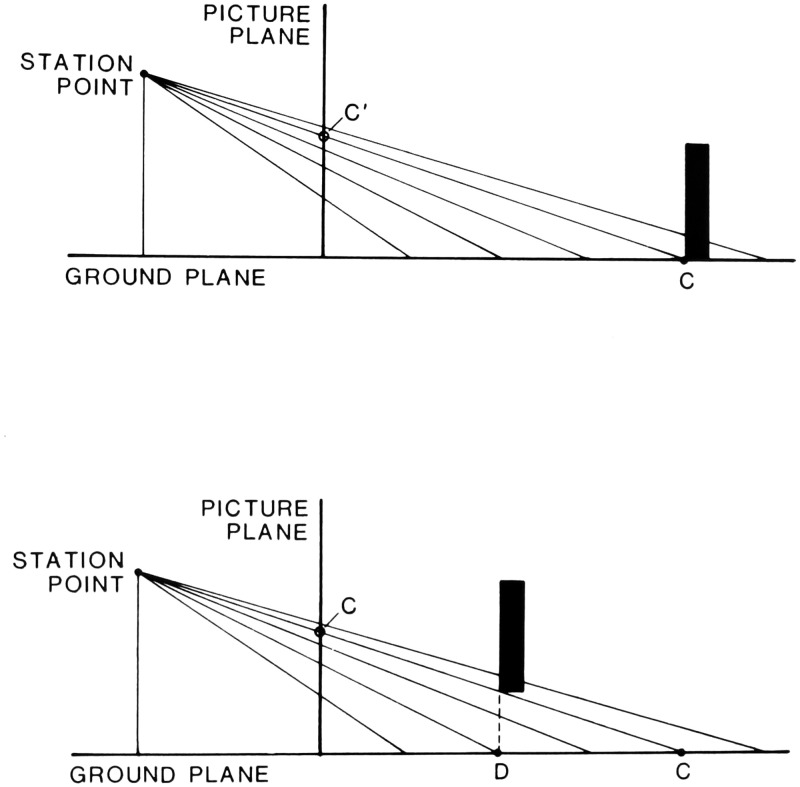
Optical contact. In the upper drawing, projective contiguity at C’ corresponds to physical contact between the object and ground at C. In the lower drawing, projective contiguity at C’ is deceptive because the object is floating in the air above D. (Adapted from Sedgwick, 1980.)

Strikingly, Gibson created an illusion that illustrates this perceptual effect. He suspended a rectangle, by means of a hidden support, above a tabletop (Figure 24). An observer then viewed the scene through a peephole placed such that the observer’s line of sight to the bottom of the rectangle extended to a farther location on the surface of the table. In this situation, as described by Gibson, the observer mistakenly saw the rectangle resting on the table at the location where the image of the base of the rectangle met the image of the table. Gibson uses the phrase “optical contact” to describe such coinciding images. He writes that “[the rectangle’s] distance is given by the optical contact of its base with the background” (p. 178).

As Gibson describes, the illusion vanishes if head motion and binocular vision are allowed. The strength of the illusion is demonstrated by the observer’s astonishment at seeing that the rectangle is actually “much smaller and suspended in the air” and also by the observation that

[e]ven with full knowledge, however, the original appearance returns, or strongly tends to do so, when monocular motionless vision is reemployed. The conclusion is that a perceived object under these conditions recedes within its cone of light rays until its surface is continuous with the background surface. (p. 180)

In spite of the centrality of the concept of optical contact to Gibson’s theory, and in spite of the strength of the illusion that he created to illustrate it, there is no further discussion of optical contact in *Visual World*. It may be that Gibson thought of his illusion as a rather special case because it used a peephole to eliminate relative motion and stereoscopic depth. Much later (Gibson, 1979, p. 159), he discusses his optical contact illusion again and asserts again that “it appears only with monocular arrested vision,” which he calls “a rare and unnatural kind of vision.” This is no doubt correct for the tabletop setup of Gibson’s illusion, in which the observer was only a few feet away from the suspended object. It is less clear how rare the optical contact illusion would be in natural settings with somewhat greater distances. The sensitivity of the optical information for depth from either motion parallax or binocular disparity decreases with the square of distance and so drops off rapidly as distance increases. Making use of Gibson’s concept of *effective stimuli*, we might modify his formulation, quoted above, to say that in the absence of *effective* relative motion or *effective* stereoscopic depth a contour is seen on the background. Also, in peripheral vision, in which much of the detail of the optic array is not available perceptually (Rosenholtz, 2016, 2020; Rosenholtz et al., 2012), the perception of physical contact may be largely based by default on optical contact. The perception of physical contact based primarily on optical contact may thus be more common than Gibson believed.

**Figure 25.**
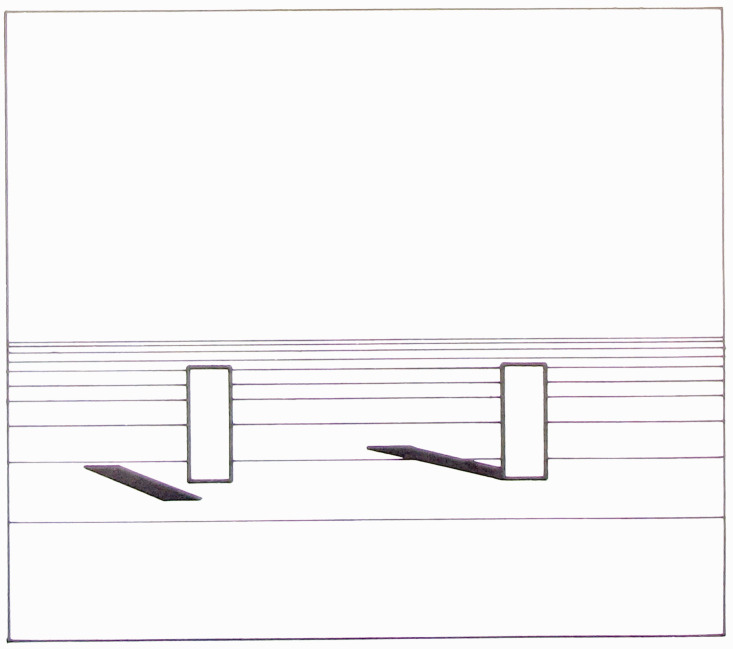
Cast shadows as information for contact. (Adapted from Sedgwick, 1980.)

In addition to motion parallax and stereo disparity, the shadow cast on the ground by an object can also affect whether or not the object is perceived to be resting on the ground (Figure 25; Mamassian et al., 1998; Yonas et al., 1978). Additional research has shown that what counts perceptually as a shadow is rather loose and need not very closely resemble what would be a geometrically accurate shadow (Ni et al., 2004).

### Ecological and Perceptual Constraints on Contact

The perceptual effect of optical contact on perceived physical contact is constrained by other perceived aspects of the spatial layout. For example, the perceived spatial orientation of a surface will constrain its contact possibilities. Gibson’s rectangle, with its parallel edges in the photograph, is perceived to be oriented vertically; thus, its contact with the horizontal surface of the table is limited to the rectangle’s bottom edge. If, however, the projected edges of the rectangle converged toward the horizon, then it could be seen as oriented horizontally and so could appear to be in contact with the ground along its whole length (Hochberg & Beck, 1954). If the available optic array information is not sufficient to specify the geographical orientation of a surface, as can happen, for example, if the shape of the surface is irregular or its angular extent is small, then the surface may by default inherit the orientation of its background surface. The interactions among a number of such constraints have been investigated computationally for some simple scenes (Sedgwick, 1987a, 1987b, 1989), showing that the 3D spatial layout of the scene can potentially be entirely determined by the interactions between environmental constraints and available optic array structures. In this model, optical contact specifies physical contact as long as this outcome is consistent with applicable constraints.

In crowded environments, an object’s contact with the ground (or other supporting surface) may be occluded, so that its location cannot be determined by optical contact. Such an object’s location relative to the ground might nevertheless be constrained by other visible surfaces; for example, the base of a floor lamp standing behind a chair might be occluded by the chair, but the location of the lamp might nevertheless be constrained by there being a wall behind it. Although the location of an object’s ground contact is not visible, due to occlusion, ground contact may nevertheless be perceived amodally, in a somewhat indeterminate location, due to the broad constraint that objects are generally supported by the ground; such perception could be called a form of perceptual completion.^21^

### Nested Contact Relations

**Figure 26.**
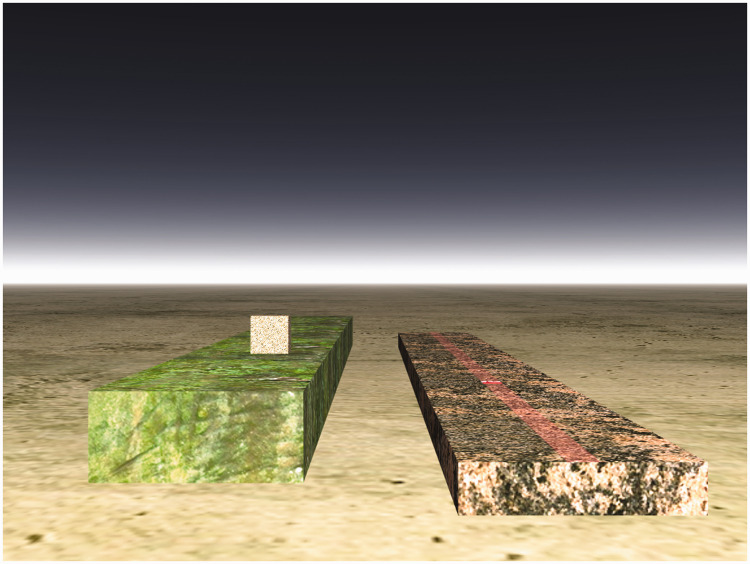
Nested contact relations. (Adapted from Meng & Sedgwick, 2002.)

In natural environments, contact relations can be quite complex. Everything that is not flying or floating is supported by the ground, but this support may be indirect. For example, an object may be resting on a table that is in turn resting on the ground. I refer to such situations as nested contact relations. Jeanette Meng and I investigated the perception of pictorial representations of such nested contact relations; in the displays we used, perceived relative locations of objects were propagated effectively through a series of nested contact relations (Meng & Sedgwick, 2001). When each of two objects was resting on a separate platform, however, and the platforms were spatially displaced relative to each other, our results suggested that “local spatial relations between objects and their platforms are only partially integrated with more global spatial relations between the discontinuous surfaces of the platforms” (Figure 26; Meng & Sedgwick, 2002).

Other related investigations have been carried out by Ni et al. (2004). The contribution of optical contact to the perception of spatial layout is a rich subject for investigation within the ground theory and is far from being exhausted.

### Non-Contact Relations

As noted above, in the discussion of Gibson’s optical contact illusion, binocular disparity and motion parallax could dispel the illusion by providing information for a separation in depth between the bottom edge of the rectangle and the surface with which it was in optical contact. Binocular disparity and motion parallax can do more, however, than simply determine whether or not there is physical contact. They also provide information about the amount of depth. Gibson says that the location in space of the rectangle, seen binocularly and with free head movements, was now seen *correctly*. Thus, the rectangle was brought into what could be referred to as a non-contact spatial relation with its background surface. A similar result, described above, was also found by Gibson and Hennemann (Gibson, 1950a, p. 183) in their exploratory observations on the interspaces between objects in a cluttered room; the spatial relations between objects not directly in contact with each other could be perceived with a considerable degree of accuracy.

Likewise, for cast shadows, the size of the gap between the image of an object and the image of the shadow it casts on a surface is an indication of the size of the physical gap separating the object from the surface. In the computer graphics displays created by Kersten et al. (1997), a sphere moving along a horizontal surface was perceived to also move up and down relative to the surface based entirely on the changing image separation between the sphere and the shadow it cast on the surface. Thus, in these situations, physical contact is the limit at which the amount of non-contact, as specified by cast shadow separation, binocular disparity, or motion parallax, goes to zero.

It might seem that the Cartesian theory, because it is based on perceived distance, could comfortably account for such non-contact relations, but it cannot. Cartesian theory is based on perceived absolute distances from the eye of the observer, whereas non-contact relations are based on perceived relative distances between surfaces.^22^ Visual perception is highly sensitive to relative depth based on either binocular disparity or motion parallax, so much so that both are referred to as hyperacuities, but it is much less sensitive to absolute egocentric distance based on stereopsis or motion. One might say that these perceptual capabilities are better adapted to the needs of the ground theory than to the needs of the Cartesian theory.

To explore the effects of background surfaces on binocular stereopsis, Barbara Gillam, myself, and our colleagues carried out experiments that compared the binocular perception of relative depth with and without a vertical background surface (Gillam et al., 1993; Gillam & Sedgwick, 1996; Gillam, Sedgwick, et al., 2011; Sedgwick et al., 2004, 2005). In one experiment, without the background surface, two luminous probes floating in empty space could be adjusted to be at the same depth with reasonable accuracy, as would be expected because this is a relative depth task. For the background surface, we exploited a weakness of stereoscopic vision that had been explored by Gillam (Gillam et al., 1984, 1988); if a vertical surface composed of random dots is slanted around a vertical axis, then the difference between the two eyes’ views is primarily that one image is horizontally compressed relative to the other. The visual system’s sensitivity to this difference in compression is limited, with the effect that the slant of the surface is perceptually underestimated. This gave us a way of assessing the perceptual effect of a background surface on the perceived relative depth of the two probes floating in space in front of the surface. If the perceptual system ignored the background surface, then it should be possible to accurately adjust the relative depth of the two probes so that they were equidistant from the observer. Alternatively, if the perceptual system made use of the background surface to determine when the probes were equidistant, then the underestimation of the perceived slant of the background would be expected to produce a systematic error in the perceived relative depth of the probes. This latter result is what we obtained. We concluded that although stereopsis can support the perception of relative depth in empty space, the perceptual system will make use of background surfaces when they are present. Other research has found related results (Glennerster & McKee, 1999; He & Ooi, 2000; Mitchison & Westheimer, 1984).

A final example of non-contact relations involves the ground surface (Wu et al., 2015). As noted above, in empty space the accurate stereoscopic perception of distance from the observer to limited to near distances. What the researchers did in these experiments was to make such measurements when the target object was floating above the ground surface. In this situation, stereoscopic relative distance could be used to identify the location on the ground above which the target floated. Then the perceived absolute distance of that location on the ground could be perceptually applied to the target object. The perceived distance of the target was substantially better when the ground was present than when it was absent. Such non-contact interaction with the ground surface might also occur for other sources of stimulus information, such as motion parallax or aerial perspective.

## Location

### Egocentric and Allocentric Perception

The relation between egocentric and allocentric space perception is an important theoretical question that is answered quite differently by the Cartesian theory and by the ground theory. Egocentric perception is relative to the self, such as the visual perception of the distance from the observer’s eye to an object. Allocentric perception is relative to something other than the self, such as the perception of an object’s location relative to the environment.

In the Cartesian theory, space perception is based on the direction and distance of points relative to the eye of the observer, so it is clearly a theory of egocentric space perception.

Gibson’s ground theory is more complex. Here is part of an earlier quote from his *Research Report*, which describes “the space in which the pilot flies”:

There is invariably beneath him a continuous terrain, and what he discriminates is the location of all points on this terrain rather than the specific distances of given points … .[T]he theory behind [this] should be a theory of continuous space with an underlying terrain in which the observer is himself located and in which he can move. (Gibson, 1947, pp. 184–185)

Implicit in this quote is the perception of a stable, stationary terrain; the observer is seen to be located in this terrain and to be moving through it. This could be a description of allocentric perception. But a little later Gibson writes: “The problem of three-dimensional vision, or distance perception, is basically a problem of the perception of a *continuous surface* which is seen to extend away from the observer” (Gibson, 1947, p. 185). The surface seen to be extending away from the observer is a description of egocentric perception. Taken together, the two quotes imply that Gibson is putting egocentric and allocentric perception together rather than contrasting them.

This linking of egocentric and allocentric perception is more explicitly stated in the *Visual World*, in a section titled “The Ego in Perception”:

Perceiving the world has an obverse aspect, perceiving oneself. Observers have often pointed out that one’s own body is represented in the visual field, and it has been argued that the ego is therefore an object in the field of experience like any other object [Koffka, 1935, pp. 319–331]. A more satisfactory statement, however, is that perceiving the environment includes the ego as part of the total process. In order to localize any object, there must be a point of reference. An impression of “there” implies an impression of “here,” and neither could exist without the other. (Gibson, 1950a, pp. 225–226)

This same idea is expressed repeatedly in Gibson’s *Ecological Approach*. For example, he titles one section “Egoreception and exteroception are inseparable” and in that section writes, quite simply, “Self-perception and environment perception go together” (Gibson, 1979, p. 116).

Although Gibson’s ground theory combined egocentric and allocentric perception from the beginning, initially both were derived from egocentric distance perception, along the ground surface, as has been discussed above. The theory initially assumed that perceived egocentric distance determined perceived size (Gibson, 1947, 1950a), and it initially used slant with respect to the line of regard (optical slant) to specify perceived surface orientation (Gibson & Cornsweet, 1952).

As we have seen, however, the ground theory gradually changed. Perceived size could be based directly on texture scale and horizon-ratio scale; these scales are allocentric in that they establish scales over the entire ground plane and do not depend upon egocentric distance (Gibson, 1959, 1979; Sedgwick, 1973). Nevertheless, the allocentric scales and egocentric distance maintain links both because of the geometrical linkage between size, distance, and visual angle (applied to perception in the form of the “size-distance invariance hypothesis”) and also because both texture scale and the horizon are also involved in forms of potential information for egocentric distance.

Likewise, it has been shown that a surface’s orientation with respect to the environment (geographical orientation) could potentially be perceived directly from the perspective structure of the surface, without depending on the surface’s local optical slants (Hay, 1974; Sedgwick, 1980), but as discussed above, geographical orientation and local optical slants are readily derived from one another. The analyses of all these forms of potential information are thus consistent with Gibson’s assertion that “egoreception” and “exteroception” are inseparable.

In the years following the publication of the *Ecological Approach*, David Marr developed a highly influential model of 3D vision (Marr, 1982), which was largely derived from the early version of Gibson’s ground theory. The initial stages of Marr’s theory assembled what he called a 2 1/2-D Sketch; Marr referred to this stage as “viewer-centered,” that is, egocentric. This sketch was composed of local surface patches, each of which had an egocentric distance and an optical slant. Marr’s interest was in object perception, so the final stage of his model was a fully 3D object, organized around its own center and axes; thus, it was allocentric. Marr referred to this stage as “object-centered.”

Based on the later developments of the ground theory, I wanted to question the assumption, built into Marr’s theory, that allocentric perception must necessarily be preceded by, and derived from, an earlier stage of egocentric perception. Because my interest was in the layout of surfaces composing an entire scene, rather than single objects, I used the term “environment-centered” rather than “object-centered” and I attempted to show, by examining the potential information in the optic array, that environment-centered perception could be obtained directly from the optic array (Sedgwick, 1983). In other words, it is not theoretically necessary to derive environment-centered perception from a prior stage of viewer-centered perception as Marr had assumed.^23^

### “Why Do Things Have Location … ?”

For my proposal that environment-centered perception need not be derived from viewer-centered perception, the horizon-ratio scale was already available for direct environment-centered size perception (Sedgwick, 1973), and the invariant perspective structure of the optic array was already available for direct environment-centered perception of surface orientation (Sedgwick, 1980, 1983), but what about the basic function of localization—the determination of where things are in the environment? Is there an alternative to the view that every point is localized by its distance (and direction) from the observer? Gibson’s concept of egocentric distance as distance along the ground from the observer was critically different from Marr’s (or Descartes’) egocentric distance from the eye of the observer, but nevertheless, I was uncomfortable with either egocentric distance or allocentric distance as a fundamental basis for perceiving a 3D layout of surfaces.

Concerning egocentric distance, I wondered what would count as the “ego” from which such distance is measured. For Gibson’s initial wartime concern with the problem of perceiving quite large distances, the answer seemed reasonably straightforward: egocentric distance would be measured along the ground from observer to object. But for shorter distances, within which an observer might be interacting with objects, the relevant starting point from which to measure distance seems less clear. It seems that the starting point—my foot, or my hand, or the tool that I am holding—changes with the tasks I might perform. Suppose I am reaching for a cup; then the diminishing distance from my hand to the cup seems most relevant; but am I then also perceiving the distances from that hand to all of the other objects near to me; and if not, then how are their positions established?

Concerning allocentric distance, my concern was that there are so many of them. Between any two visible locations in the scene there is an allocentric distance: the dense web of distances between each object in my visual field and every other object, or perhaps the much denser web of all the distances between every discernible point on every object and surface. Yet, at any given time only a few, if any, of that vast number of distances will be of interest to me. If perception is an activity, it takes energy; that seems like a lot of wasted energy.

It was reflections of the above kind, as I attempted to model environment-centered perception of spatial layout, that led me to wonder if distance—egocentric or allocentric—might be the wrong basis for localizing everything in my visual field. Perceived distances seemed most useful when required for the particular actions in which the organism is currently engaged.

But if not by distance, then how are things localized on a surface?

Near the beginning of *Visual World*, Gibson asks “Why do things have location, that is, how can we see where they lie?” (Gibson, 1950a, p. 2). A little later, he begins to answer this question: “Some [surfaces] have closed contours or shapes and they are located with reference to the ground” (p. 4). Still later he writes, “Let us assume that an object is seen where its contour interrupts the background—at that distance and no other—except when depth-at-a-contour brings it forward in distance” (p. 177). He describes the object’s location on the background surface as “the point to which it is attached” (p. 181).

In 1950, Gibson assumed that the object’s point of attachment needed to be further localized by specifying its egocentric distance from the observer, but I suggest that this is not necessary to a more fully developed ground theory. An object’s point of attachment to the surface of support is usually unique to that object and is usually sufficient to locate that object within its environment. In my proposal that the environment-centered perception of spatial layout need not be derived from a prior viewer-centered stage of perception, an object’s location is specified by its contact relations within the layout of surfaces comprising its environment (Sedgwick, 1983, 1987a, 1987b).^24^

### Environment-Centered Location

Here I propose a baker’s dozen characteristics of environment-centered location:

1. Environment-centered (allocentric) location need not be derived from viewer-centered (egocentric) location.
2. Environment-centered location is defined by contact relations within the layout of surfaces comprising the environment.
3. Environment-centered location is a primitive of the visual perception of spatial layout.
4. Environment-centered location perception is usually quick, accurate, and effortless.
5. Environment-centered location of an object is invariant under movement of the observer. Thus, it needs no updating as the observer moves (unless the object actually moves). What needs updating is the location of the observer in the environment.
6. Environment-centered location applies equally to objects and to features of the environment, such as a hill or a hole in the ground.
7. Environment-centered location applies to every level of the hierarchy of contact relations (e.g., a table is located on the floor, a book is located on the table, etc.).
8. Environment-centered location is, as its name implies, local. An object’s contact relations only need to be determined for its level of the hierarchy of contact relations (e.g., the book’s location on top of the table); the object’s spatial relations to lower levels in the hierarchy (e.g., to the floor) are inherited.
9. Environment-centered location is singular; that is to say, at any given time an object has only one location (although this location can complexly hierarchical). This is in contrast to allocentric distance; an object in a rich environment simultaneously has a great many allocentric distances, including those between each of the other locations in the environment.
10. Environment-centered location can enter into any of a rich set of spatial relations among other objects in contact with the same surface or related surfaces. Among those are:
  - adjacency (sharing an edge, a corner, or a point of tangency)
  - alignment or collinearity
  - surrounding or surrounded by
  - partially enclosing or partially enclosed by
  - supporting or supported by
  - transitivity between levels of the contact relations hierarchy
11. Environment-centered location is invariant under various transformations of perceived space, such as affine or perspective transformations. This is another sense in which location is invariant under movement of the observer. As noted above, efforts to map perceived allocentric distances within a portion of a ground surface have found that the depth component of distances is somewhat compressed relative to the width component. Thus, perceived allocentric distances change, compressing or decompressing, when the observer moves to another viewpoint, in which the depth and width of the mapped area are exchanged. This has raised the question of why an observer moving through an environment does not see continual deformation produced by all those changes in allocentric distances. The puzzle is solved, however, if it is the location of each object (not its web of allocentric distances) that is perceptually salient to the observer; the object’s point of contact with the environment is invariant under such transformations of allocentric (or egocentric) distances.
12. Environment-centered location is usually phenomenologically readily accessible and expressible (e.g., Q: “Hey guys, does anyone know where my car keys are?”; A: “Sure, they’re in the kitchen, on top of the microwave”). This is in contrast to coordinate systems based on distance and direction (either egocentric or allocentric); I suggest that accessing and expressing an answer to the above question as coordinates would be difficult for untrained observers except in the simplest situations (e.g., A: “Sure, they’re right in front of your nose”). Although where an object is can be accurately expressed either as a location or as set of coordinates, I suggest that these two systems are incommensurable, in the sense of the term used by Thomas Kuhn (1982).
13. Environment-centered location is readily communicable, as in the above example, in contrast to egocentric distance and direction. An answer in terms of the object’s location depends neither on the location of the questioner nor on the location of the answerer. Giving an answer in terms of egocentric distance and direction is trickier. If the answerer expresses the distance and direction of the object from their own point of view, then the questioner will also need to know the coordinates of the answerer and to then use that information to transform the answer into the questioner’s point of view (alternatively, the answerer could make the transformation, but that is no easier overall).

The concept of environment-centered location, in its interactions with other optic array information for spatial layout, has been formally investigated using an expert system (Sedgwick, 1987a, 1987b, 1989), and a few empirical investigations have been carried out (Meng & Sedgwick, 2001, 2002), but many of the above propositions are at present only supported by the writer’s phenomenology. As with contact relations, environment-centered location offers many opportunities for investigation.

### Extension

Is location enough? Without a dense web of perceived distances keeping everything in its place, what keeps the 3D layout of surfaces from collapsing or becoming indeterminate, from losing its essential three-dimensionality? My tentative answer is that a fundamental perceptual quality of a continuous surface is *extension*. This extension is a perceptual quality belonging to the entire continuous surface. It underlies point-to-point distances but is distinct from them.

In his 1947 *Research Report*, Gibson seems to be trying out various ways of describing a perceived spatial quality of surfaces that is different from conventional distance. In the space of a few pages, he says repeatedly what he is *not* talking about:

the specific distances of given points (p. 184)

the ability to judge the distances of a number of specific objects (p. 185)

almost never a single distance (p. 185)

not the “cues” or “indicators” to the distances of specific objects (p. 186)

At the same time, he seems to be searching for a way of expressing what he *is* talking about:

a continuous terrain (p. 184)

a continuous space (p.185)

a dimension of distance (p. 185)

an extended ground surface (p. 185)

continuous distance (p. 186)

the dimension or sensory continuum of distance, *as such*, which, once visible, determines how distant all the objects within it are (p. 186)

It seems quite possible to me that Gibson is trying to get at the perceived surface quality that I am here calling extension, but I cannot be sure because he does not elaborate on the terms he is using. As I am using it, the concept of extension implies distances; an extended continuous surface has implicit in it a dense web of distances; but perhaps none of those particular distances are present in perception if they are not explicitly attended to or needed as part of the activity of the observer.

Within ground theory, other surface characteristics interact with and support *extension*. The *geographical slant* of a surface, combined with its *contact relations* may specify for perception how that surface fits into, and is *scaled* in relation to, a layout of other extended surfaces. An absolute scale factor would be established by the body scale of an observer located in this scene. Finally, a nested hierarchy of contact relations among smaller and larger objects and surfaces would establish the locations of these objects and surfaces within the overall scene.

### Action in a Stable World

So, what now is the role of distance perception in the ground theory? The answer, I believe, lies in action. When individuals act in the world, then it seems clear that “the specific distances of given points” are necessarily involved in many such actions. Such perceived distances may often be highly specific to an action, such as throwing the crumpled piece of paper in my hand into the wastebasket in the corner. In this example, the distance is egocentric, but actions can also involve allocentric distances; for example, I might be tidying up the classroom and trying to arrange the chairs so that they are equally spaced. The perception of such distances, and perhaps of most distances, would serve no purpose outside of the particular actions that require them. Gibson said something analogous in his 1947 *Research Report*: “It might be suggested that the practical value of depth or distance perception is that it makes possible locomotion through a continuous space which includes obstacles … ” (p. 185).

Gibson (1979, pp. 127–143), introduced a new term, “affordance,” to connect perception to action. Affordances are perceptual information supporting our actions in the world. In a complex natural environment, in a given stretch of time, there are almost unlimited possible actions, just as there are almost unlimited distances, and the potential information linking some of these actions with the appropriate distances is, according to Gibson, available in the transforming optic array. I am suggesting that it is only when we are involved with particular activities—whether in performing them or in some other way, such as planning or imagining them—that we perceive the relevant affordances and the particular distances associated with those affordances.

Currently, many researchers are attempting to better understand the neurophysiology underlying egocentric perception, allocentric perception, the transformations between them, and their relation to action in the world. J. J. Gibson’s ground theory of space perception might be of use in this effort, which might in turn led to further developments of the ground theory.^25^

## Notes

1 Because this paper is primarily about the work of J. J. Gibson, the name “Gibson,” without initials, will be understood throughout to refer to him. Initials will be used when needed to distinguish between J. J. Gibson and E. J. Gibson.

2 For clarity, I have altered Olscamp’s translation of “position,” referring a position on the back of the eye, to “angular direction,” which Descartes assumes would be obtained from that position.

3 What is of most relevance to Gibson’s developing ground theory is contained in several sections of Chapter 9 of his report. A footnote at the beginning of that chapter reads “This chapter was written by the editor in collaboration with N. M. Glaser.” I know of no additional information concerning Glaser’s contribution. Gibson’s greatly enlarged exposition of the ground theory (Gibson, 1950a) mentions a few other members of his air force group, but it does not mention Glaser.

4 I thank Diederick Niehorster for drawing my attention to Gibson’s 1957 talk and providing me with a copy of it.

5 Because Gibson’s books are frequently mentioned here, after the first instance of each full title, the following italicized abbreviated titles will be used: *Motion picture testing and research: report no. 7 Research Report* *The perception of the visual world Visual World* *The senses considered as perceptual systems Senses Considered* *The ecological approach to visual perception Ecological Approach*

6 Gibson’s *Research Report* notably also introduced optical flow fields as a critical component of his theory, but that aspect of his work is not covered here; it is discussed in detail elsewhere in this special issue (Rogers, 2021; Warren, 2021). Gibson’s concept of texture was always about natural textures, with all their variety, but he lacked the tools with which to study them; recent advances in studying the perception of natural materials are also discussed in this special issue (Todd, 2020).

7 Gibson made this point again in *Ecological Approach*. Looking back, he wrote “I would now describe the ground theory as a theory of the *layout* of surfaces. By *layout*, I mean the relations of surfaces to the ground and to one another, their arrangement” (Gibson, 1979, p. 148).

8 Gibson’s 1957 talk, referred to above, also uses the term “optical array.” A long abstract of the talk was published in 1959 in the proceedings of the conference in which the talk had been presented. Taking the lag in publication times into account, Gibson’s actual writing of this 1959 chapter, probably took place at roughly the same time (1957) as the writing of the 1957 talk. He is clearly in the process of developing new concepts and trying out names for them. His 1957 talk, which uses the term ecological optics, primarily uses what seems intended as an equivalent term: “environmental optics.”

9 The 1957 talk also includes a clear description of the concept of “potential information.” As noted above, Gibson’s chronological order of writing the 1957 talk and the 1959 chapter is not known; Gibson may also have been willing to go farther in a talk than in a publication.

10 Among important components of Gibson’s ground theory that are not discussed here are non-planar surface shapes generally, and 3D object shapes in particular; edges; occlusion (including dynamic occlusion) and transparency.

11 In addition to angle of elevation in the array, the cylinders’ angles of declination from the implicit horizon also provides potential information for their relative distances (Messing & Durgin, 2005; Ooi et al., 2001; Sedgwick, 1973, 1980, 1983).

12 An example of this distinction between absolute and relative scale is Gibson’s experiment on size-matching with an extended ground plane (Figure 5). The experiment was field-tested with observers who were actually outdoors, looking at the stakes; the scene was then photographed with standard stakes of various heights at various distances, and these photographs were used to develop a “distance estimation” test that could be administered in a classroom setting. Because the test task required observers to choose which comparison stake matched the height of the standard stake, this task could be done about as well using relative scale photographs as in the absolute-scale field-testing situation.

13 The motivation for this experiment was to contrast elevation in the visual field with a more general formulation of the potential information in these two situations (floor or ceiling contact), which is that in both cases perceived distance increases as angular distance from the observer’s horizon decreases (Sedgwick, 1973, 1980, 1983). This more general formulation is still contingent for its validity on the object’s contact with the relevant surface.

14 Only the special case of 0 deg optical slant (i.e., no slant) is tested. The tasks in the two conditions were not equivalent.

15 Although optical slant is in some ways suited to the visual field, it is a somewhat uncomfortable fit because each local surface patch is slanted in depth, whereas the visual field is conceptually flat. David Marr, who took much from Gibson (but also missed much), invented for optical slant what he called the 2 1/2-D sketch, placing it in a conceptual position midway between the flat image and the fully 3D object (Marr, 1982).

16 An augmented version of Hay’s ghost image, described below, has also been used for the same purpose (Sedgwick, 1991, 2003).

17 Although Purdy’s report is dated 1960, it was submitted as his dissertation in 1958.

18 A great circle on the surface of the sphere is a circle whose plane passes through the center of the sphere; if the optic array is considered as a sphere, then the sphere’s center is the point of observation.

19 It was precisely this “mistake” that Marr (1982), relying on Gibson’s earlier work, made the basis of his theory of vision.

20 There are, however, pre-Cartesian formulations of the perception, or representation, of visual space for which contact is a distinct issue, specifically the theoretical traditions connected with Alhazen (Sedgwick, 2001a) or Alberti (Sedgwick, 1980, 2001b), respectively.

21 I am indebted to Diederick Niehorster for suggesting this line of thought.

22 Binocular stereopsis was not known to Descartes, but after Wheatstone’s discovery of it, it was quickly assimilated into the Cartesian tradition. Gillam has pointed out that Wheatstone’s original demonstrations of stereopsis contained far more complexity and higher-order information than the highly reductive experimental investigations that followed (Gillam, 1995).

23 Marr’s theory also imported from computer programming the then new design concept of modularity, reifying the list of cues to egocentric distance as semi-independent neural modules (for stereopsis, motion, shading, etc.) whose outputs were postulated to be combined to obtain the egocentric distances of surface patches. Barbara Gillam and I have argued elsewhere that a modular theory such as Marr’s is neither the most accurate nor the most fruitful approach to understanding visual space perception (Sedgwick & Gillam, 2017).

24 Loomis and his colleagues have made extensive use of the term “location” in theorizing about measuring spatial perception and updating, but their definition of location is egocentric, based on perceived egocentric direction and distance (reviewed in Loomis & Philbeck, 2008).

25 This article is based on a presentation given at the 2019 ECVP symposium entitled “The Ecological Approach of James J. Gibson: 40 years later” (Sedgwick, 2019).
